# Protein Extraction from *Chlorella pyrenoidosa* Using *Bacillus* spp. Isolated from *Jeotgal*: Strain isolation, Characterization, and Fermentation

**DOI:** 10.4014/jmb.2411.11070

**Published:** 2025-05-15

**Authors:** Kyung-Jin Cho, Min-Ung Kim, Geum-Jae Jeong, Do Kyung Oh, Ju-Hong Kang, Da-Hyeon Yoon, Fazlurrahman Khan, Young-Mog Kim

**Affiliations:** 1 Department of Food Science and Technology, Pukyong National University, Busan 48513, Republic of Korea; 2 Research Center for Marine Integrated Bionics Technology, Pukyong National University, Busan 48513, Republic of Korea; 3 Ocean and Fisheries Development International Cooperation Institute, Pukyong National University, Busan 48513, Republic of Korea; 4 International Graduate Program of Fisheries Science, Pukyong National University, Busan 48513, Republic of Korea

**Keywords:** *Jeotgal*, *Bacillus* spp. fermentation, *Chlorella pyrenoidosa*, protein extraction

## Abstract

*Chlorella pyrenoidosa*, a versatile microalga with a rich nutritional profile and functional components, has various applications. However, rigid cell walls pose challenges for the effective extraction of proteins. Microbial fermentation is a promising solution for large-scale production and industrial applications. This study aimed to isolate *Bacillus* spp. with high enzymatic activity from *Jeotgal*, a Korean traditional fermented food, and enhance protein extraction from *C. pyrenoidosa* using microbial fermentation with the isolated *Bacillus* spp. Twenty-two strains of *Bacillus* spp. were isolated, and eight *Bacillus* species were selected based on their ability to produce cellulases, proteases, and lipases. Microbial safety was further assessed by testing for biogenic amine production and hemolytic activity. All eight strains exhibited γ-hemolysis, with four strains not producing biogenic amines. Notably, fermentation using *Bacillus amyloliquefaciens* F2 showed the highest protein extraction yield at 35.45 ± 1.21% (v/v). In conclusion, this study demonstrates the potential of microbial fermentation for protein extraction from *C. pyrenoidosa*, offering a novel approach for its utilization in the food industry.

## Introduction

As the interest in sustainable food sources increases, marine biomass is emerging as a nutritious alternative to existing food materials. Marine biomass refers to all ocean organisms that can be utilized as resources. It has been reported that they are beneficial to the human body and emit less carbon dioxide [1, 2]. *Chlorella pyrenoidosa* (*C. pyrenoidosa*) is a green microalga containing various minerals (magnesium, calcium, iron, and potassium), vitamins, omega-3 fatty acids, and essential amino acids. In particular, *C. pyrenoidosa* has a high protein content and has attracted attention as a future food resource [3, 4]. However, it has a rigid cell wall, which makes the digestion and absorption of nutrients difficult. Physical methods such as ultrasonication [5], sonication [6], freeze-thawing [7], and high-pressure treatment [8], and chemical methods such as organic solvent extraction [9], salt precipitation [10], and enzyme treatment [11], have been used to extract proteins from *C. pyrenoidosa*. Microbial fermentation is carried out at low temperatures and pressures, offering advantages such as reduced ingredient denaturation, lower energy consumption and costs compared with other extraction methods, and minimal environmental impact [12]. Owing to these advantages, microbial fermentation has been used to extract plant proteins. Dai *et al*. (2022) effectively extracted proteins from soybean meal by fermentation with *Bacillus licheniformis* and *Bacillus subtilis* [13], resulting in an increase in the phenol and pyrazine contents. Xiao *et al*.(2009) extracted crude proteins from macroalgae via fermentation with *Aspergillus niger* and *Candida utilis* [14].

*Bacillus* species are among the most important industrial microorganisms that produce cellulases, proteases, lipases, and glucanase [15, 16]. In addition, *Bacillus* spp. have been reported to exhibit functional properties, such as antioxidant and probiotic activities [17-19]. Many studies have been conducted to isolate *Bacillus* spp. from traditional Asian fermented foods such as *Cheonggukjang*, *Natto*, *Tempeh*, and *Jeotgal* [20-24]. *Bacillus* spp. isolated from *Jeotgal*, a traditional Korean high-salinity fermented food, possess various enzymatic systems capable of breaking down complex biomolecule [25, 26]. This study aimed to isolate *Bacillus* spp. from *Jeotgal* exhibiting exceptional enzymatic activities and to assess their safety and antimicrobial activity. Additionally, we evaluated the potential of these *Bacillus* isolates as a biological resource for protein extraction from *C. pyrenoidosa*.

## Material and Methods

### Materials and Microorganisms

Three types of *Jeotgal* (flounder, squid, and anchovy) were purchased from the Jeju Dongmun Market in Korea. *C. pyrenoidosa* powders were obtained from Jeongwoodang Co., Ltd., (Republic of Korea). All other chemicals and reagents were of analytical grade and included the following: L-histidine, L-lysine, L-ornithine monohydrochloride (L-ornithine), L-arginine, L-tyrosine, and carboxymethyl cellulose (CMC) from Sigma-Aldrich (USA), sodium chloride (NaCl) and D-glucose from Junsei Chemical Co., Ltd., (Japan), and bromocresol purple, congo red, pyridoxal 5-phosphate monohydrate, and mineral oil from Duksan Pure Chemicals Co., Ltd., (Republic of Korea). *Escherichia coli* KCTC 2571, *Bacillus cereus* KCTC 1092, *Staphylococcus aureus* KCTC 3881, and *Listeria monocytogenes* KCTC 3569 were obtained from the Korean Collection for Type Culture (KCTC, Republic of Korea). *Pseudomonas aeruginosa* KCCM 11321 was obtained from the Korean Culture Center of Microorganism (KCCM, Republic of Korea). These bacteria were maintained in glycerol stocks at -80°C and used in the experiment

### Isolation of *Bacillus* spp. from *Jeotgal*

The isolation of *Bacillus* spp. from *Jeotgal* was carried out using previously described procedures with some modifications [27]. *Jeotgal* (25 g) was combined with 225 ml of 0.1 M phosphate-buffered saline PBS (pH 7.2) and homogenized in a stomacher (BagMixer 400 W; Interscience, France) for 4 min. The suspension was serially diluted and inoculated onto all culture agar (ACA, Sigma-Aldrich) to isolate the bacteria. The inoculated ACA plates were incubated aerobically and anaerobically at 37 ± 1°C for 24 ± 2 h. Colonies were selected based on their morphological characteristics such as size, color, shape, and presence of bubbles. The colonies were streaked onto ACA plates and incubated under the same conditions. This process was repeated until a single colony was formed. The isolated strains were stored in a glycerol stock at -80°C and used in the experiment.

### DNA Extraction, PCR Amplification and Identification

DNA was extracted from a single colony using a DNA extraction kit (Bioneer Co., Ltd., Republic of Korea). Subsequently, polymerase chain reaction (PCR) was performed using the extracted DNA, AccuPower PCR premix (Bioneer), and universal primers (27F, 5’-AGAGTTTGATCTGGCTCAG-3’; 1492R, 5’-TACGGTTAC CTTGTTACGACTT-3’). The PCR protocol consisted of 30 cycles: denaturation at 94°C for 1.5 min, annealing at 55°C for 0.5 min, and extension at 72°C for 4 min in a PCR thermal cycler (Takara, Republic of Korea). The 16S rRNA sequences of the PCR products were analyzed for molecular biological identification. The sequences were edited and assembled into contigs using BioEdit Sequence Alignment Editor (Table S1). All contigs were compared against nucleotide sequences in the GenBank database (https://blast.ncbi.nlm.nih.gov/Bl-ast.cgi) using the Basic Local Alignment Search Tool (BLAST) search algorithm. Strains were identified by selecting those showing the highest sequence similarity (at least 99.80%) to the previously reported standard strains.

### Enzyme Activity

The enzymatic activities of *Bacillus* spp. were measured for protease, cellulase, and lipase. All enzyme activities were determined by comparing the size of the clear zone formed after incubation with the colony or agar well size (Fig. S1A), using the following formula:

Enzyme activity (ratio) = Clearance zone size (mm) / Colony size (mm)

### Protease Activity

The Protease activity was analyzed according to a previously described method [28]. *Bacillus* spp. was inoculated into ACA supplemented with 1% (w/v) skim milk (BD Difco, USA). Subsequently, the inoculated plates were incubated at 37°C for 24 h.

### Cellulase Activity

Cellulase activity was analyzed using carboxymethyl cellulose (CMC) agar as described by Liang *et al*. (2014)[29]. CMC agar was prepared with the following medium composition: peptone 10 g/l, CMC 10 g/l, MgSO_4_ 0.2 g/l, NaCl 0.5 g/l, CaCl_2_ 0.1 g/l, Agar 15 g/l dissolved in distilled water. Then, the medium was sterilized by autoclaving at 121°C for 15 min. *Bacillus* spp. were inoculated onto CMC agar and incubated at 37°C for 48 h. Then, the plates were stained with 1% (w/v) Congo red solution for 15 min and destained with 1 M NaCl solution for 15 min

### Lipase Activity

The lipase activity was assessed using a primary plate-screening assay [30]. Agar plates were prepared with the following medium composition: 20 g/l agar, 20 g/l Tween 80, and 0.1 g/l methyl red dissolved in distilled water. Agar wells with a diameter of 0.5 cm were made in each plate using a sterilized microtip. The strains were cultured in ACB at 37°C for 24 h and centrifuged at 13,000 g for 15 min (Hanil Supra R22; Republic of Korea). A 25 μl aliquot of the supernatant was inoculated into the agar wells and cultured at 37°C for 24 h.

### Antibacterial Activity

The antibacterial activity of *Bacillus* spp. isolated from *Jeotgal* was evaluated against five pathogenic bacteria using a modified Kirby-Bauer assay [31]. Each pathogen and *Bacillus* spp. were inoculated separately into Muller Hinton broth (MHB, BD Difco) and cultured at 37°C for 24 h. The pathogen culture, initially at a concentration of 10^8^ CFU/ml, was diluted to 10^5^ CFU/ml using MHB and spread on Mueller Hinton agar (MHA, BD Difco). The pour plate method was employed to determine the concentration of viable cell count. Then, 100 μl of cultured *Bacillus* spp. was inoculated onto a paper disk (8 mm; ADVANTEC, Japan) and aseptically transferred to an MHA plate. The MHA plate was incubated at 37°C for 24 h, and the diameter of the inhibition zone was measured in mm. Antibacterial activity was determined by comparing the size of the clearance zone formed after incubation with the colony size (Fig. S1B) using the following formula:

Antibacterial activity (ratio) = Clearance zone size (mm) / Colony size (mm)

### Safety Evaluation of *Bacillus* spp.

Hemolytic activity and biogenic amine productivity were analyzed to assess the microbial safety of *Bacillus* spp. isolated from *Jeotgal*. Hemolytic activity was determined by examining the clearance zone of ACA containing 5%sheep blood (v/v; KisanBio) [32]. The *Bacillus* spp. were plated on ACA and incubated at 37°C for 24 h. Biogenic amine productivity was analyzed for histamine, cadaverine, tyramine, and putrescine according to the method described by Yilmaz *et al*. (2022) [33]. The decarboxylase broth was prepared by adding pyridoxal 5-phosphate (5 mg/l) and 1% L-arginine, L-tyrosine, L-histidine, or L-lysine. *Bacillus* spp. were inoculated into each prepared broth and incubated anaerobically at 37°C for 24 h. The medium change from yellow to purple was determined as positive.

### Fermentation of *C. pyrenoidosa* for Protein Extraction

*C. pyrenoidosa* was suspended and fermented with the isolated *Bacillus* spp. to assess the enzymatic activity of each strain. Initially, 5% (w/v) *C. pyrenoidosa* medium was prepared by mixing 10 g of *C. pyrenoidosa* powder with 200 ml of distilled water. Subsequently, 1% (v/v) of each *Bacillus* strain, at a concentration of 10^7^ CFU/ml, was inoculated into the medium and cultured at 37°C with shaking at 150 rpm for 24 h. The pour plate method was employed to determine the concentration of *Bacillus* strains. A control experiment (without inoculation) was also conducted under identical conditions. The culture medium was then centrifuged at 10,000 g for 20 min to separate the supernatant, which was used to compare the protein extraction yields. The protein concentration was determined using the bicinchoninic acid (BCA) assay, employing the Pierce BCA Protein Assay Kit (Thermo Fisher Scientific, USA), as described earlier [34]. A standard curve was generated for protein quantification using bovine serum albumin. The protein yield from *C. pyrenoidosa* was calculated using the following equation:

Protein extraction yield (%) = P1/P0 × 100%

Where P1 is the protein content of the fermented *C. pyrenoidosa* extract (mg/ml) and P0 is the protein content of the uninoculated *C. pyrenoidosa* medium (mg/ml).

### Statistical Analysis

All experiments were conducted in triplicates. The results were analyzed using SPSS 27 (Statistical Package for Social Science, USA) to calculate the mean and standard deviation of each test group and Duncan’s multiple range test. Statistically significant differences were observed at the 5% significance level (*p* < 0.05).

## Results and Discussion

### Identification of Isolated *Bacillus* spp.

**Fig. 1:**
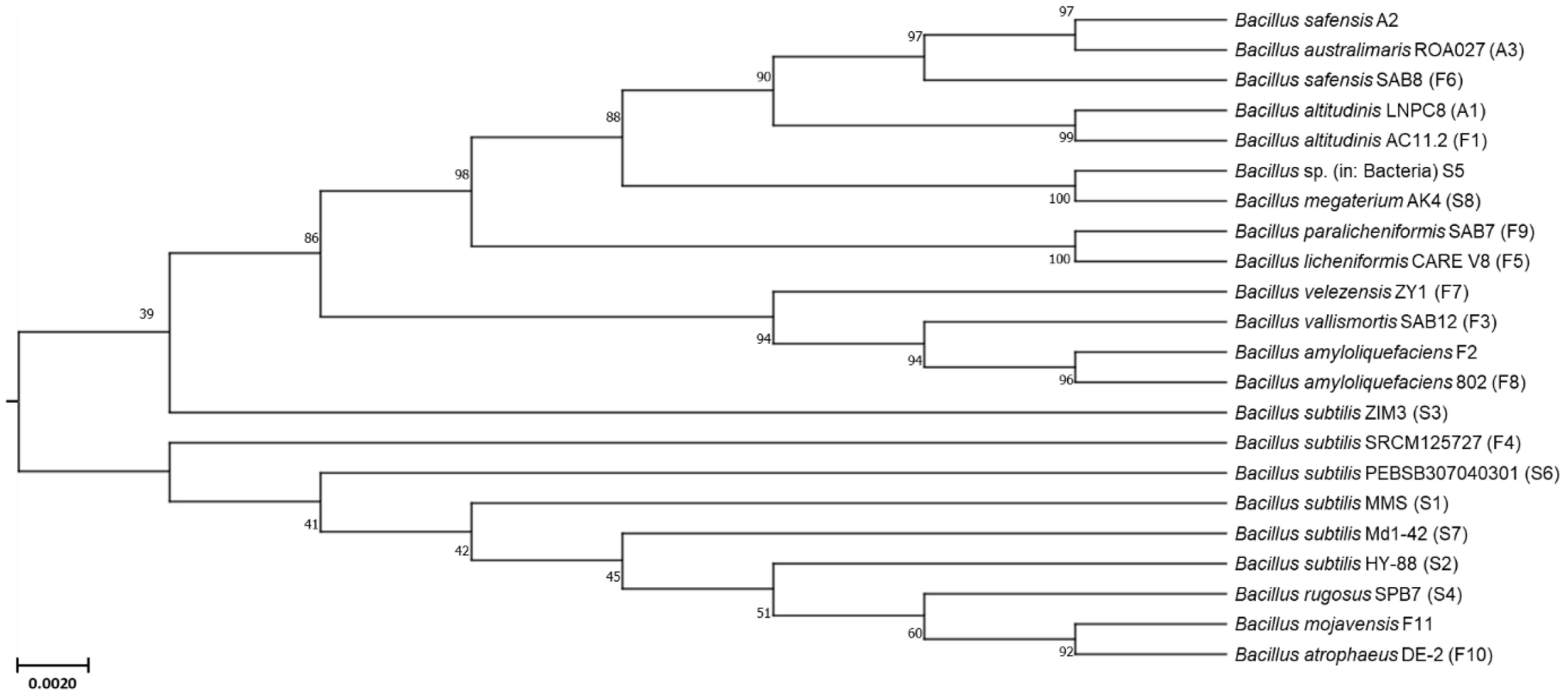
Phylogenetic tree of *Bacillus* spp. isolated from *Jeotgal* products. Evolutionary analyses were conducted using the MEGA11 software, based on 16S rRNA sequences using the maximum likelihood method. Bootstrap values (1,000 re-sampling) are shown at the branch point. The final dataset comprised 1,076 positions.

The identification results for twenty-two *Bacillus* spp. isolated from the three types of *Jeotgal* are shown in Table 1. Eleven *Bacillus* strains were isolated from flounder *Jeotgal*, eight from squid *Jeotgal*, and three from anchovy *Jeotgal*. The 16s rRNA gene sequences of the twenty-two isolates were identified as follows: *B. subtilis* (six species), *B. altitudinis* (two species), *B. amyloliquefaciens* (two species), *B. safensis* (two species), *B. megateruim* (two species), *B. licheniformis* (one species), *B. paralicheniformis* (one species), *B. rugosus* (one species), *B. atrophaeus* (one species), *B. mojavensis* (one species), *B. vallismortis* (one species), *B. velezensis* (one species), *B. australimaris* (one species), and *Bacillus* sp. (unknown; one species), and were named F1-F11, S1-S8, and A1-A3. A phylogenetic tree was constructed to represent genetic relationships among the twenty-two isolates (Fig. 1). The phylogenetic tree was drawn to scale, and branch lengths were expressed in the same units as the evolutionary distance used to infer the phylogenetic tree [35]. Guan *et al*. (2011) analyzed bacterial communities in anchovy and shrimp *Jeotgal* and identified various *Bacillus* spp., suggesting that these bacteria play an important role in fermentation [36]. In addition, it has been reported that *Bacillus* spp. isolated from high-salinity *Jeotgal* exhibit high protease activity [37, 38]. Yin *et al*. (2024) found that an environment with 10% salinity enhanced the secretion of extracellular polymeric substances (EPS) from *Bacillus* spp. with notable expression of the *DegS* gene [39]. *DegS* is crucial for activating the expression of the *aprE* gene, which is involved in the production of alkaline proteases. Consequently, *Bacillus* spp. isolated from high-salt *Jeotgal* are well-suited for decomposing marine biomass, such as *C. pyrenoidosa*.

### Selection and Evaluation of Enzyme Activities of Isolated *Bacillus* spp.

**Fig. 2:**
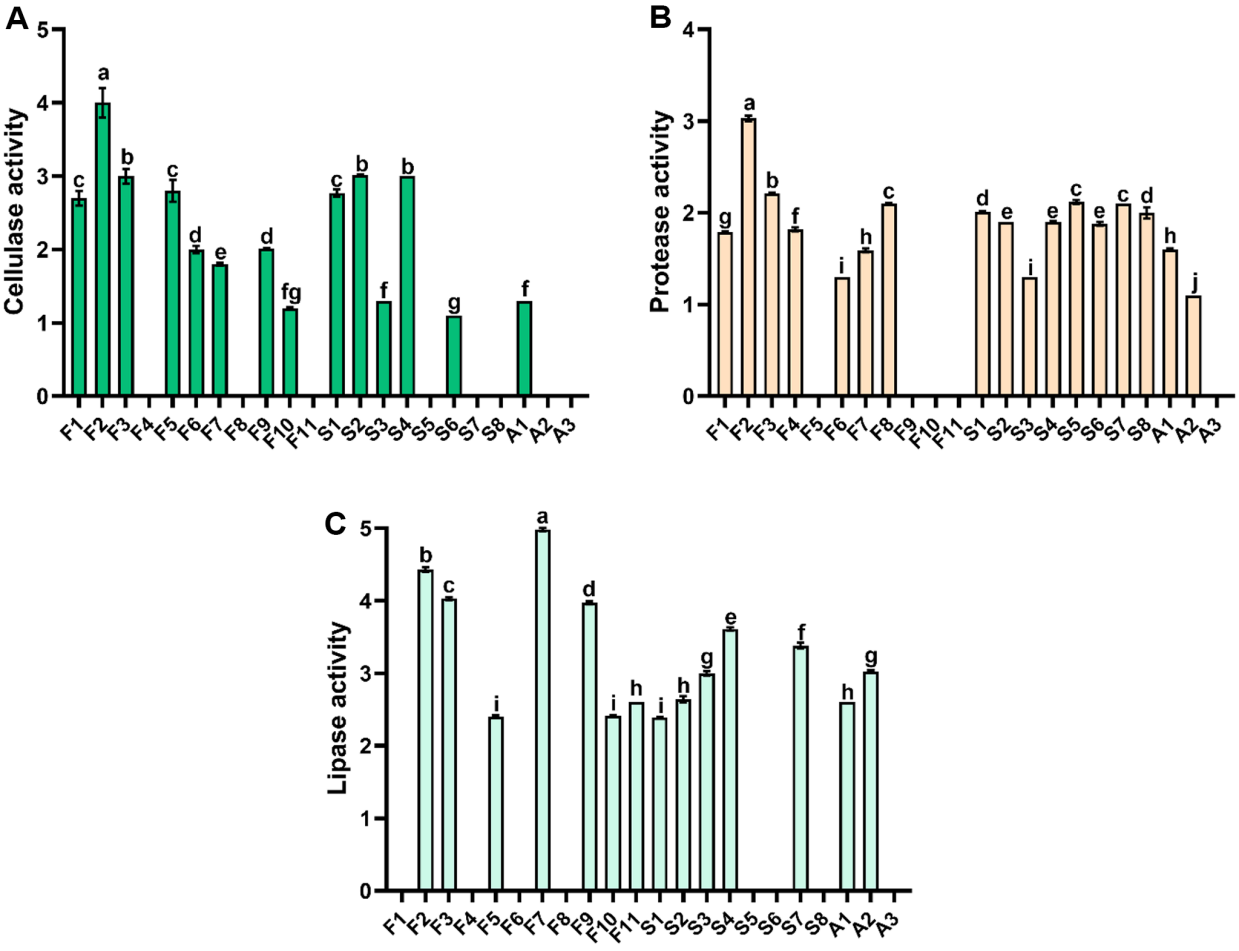
Analysis of three enzymatic activities of isolated *Bacillus* species. (**A**) Cellulase activity, (**B**) protease activity, (**C**) lipase activity. Enzyme activity was calculated as the clearance zone size (mm) divided by the colony size (mm). Values with different letters vary significantly (*p* < 0.05).

The enzymatic activities of *Bacillus* spp. isolated from *Jeotgal* are shown in Fig. 2. Among the twenty-two *Bacillus* strains, eighteen strains showed cellulase activity, fourteen strains showed protease activity, and fourteen strains showed lipase activity. All strains showed enzymatic activity for at least one substrate and eight strains (F2, F3, F7, S1, S2, S3, S4, and A1) showed enzymatic activity for all three substrates. In particular, strain F2, which was identified as *B. amyloliquefaciens*, showed the highest enzyme activity, with values of 4.00 ± 0.20 for protease, 3.03 ± 0.03 for cellulase, and 4.43 ± 0.03 for lipase. *B. amyloliquefaciens* is a representative cellulolytic bacterium known to have high cellulase activity and to produce various hydrolytic enzymes, such as lipase, α-amylase, and protease [40-43]. In addition, *B. amyloliquefaciens* produces hydrolytic enzymes in the food industry due to its thermostable enzyme productivity [44]. Zhang *et al*. (2023) previously reported the use of *Bacillus* spp. as microbial starters to extract proteins from *C. pyrenoidosa* [45]. In addition, Kim *et al*. (2012) reported that the enzyme activities of *Bacillus* spp. varied depending on the source of isolation [46]. Thus, the eight *Bacillus* spp. isolated from *Jeotgal*, which possess various enzyme systems (including cellulase, protease, and lipase activities), could serve as microbial starters capable of breaking down complex biomolecules such as *C. pyrenoidosa* [25].

### Evaluation of the Antibacterial Activity of Eight of *Bacillus* spp. Possessing Various Enzymatic Activity

During fermentation, the antibacterial properties of *Bacillus* spp. are crucial for inhibiting the growth of harmful pathogens, thereby enhancing food safety and ensuring consistent fermentation quality [47]. Table 2 presents the antibacterial activities of the eight *Bacillus* strains against the five types of pathogens. All the selected *Bacillus* spp., except S1, demonstrated antibacterial activity against *L. monocytogenes*. Notably, strains F2, F7, and S4 exhibited antibacterial effects against *S. aureus*, with F2 exhibiting the strongest activity. Some *Bacillus* spp. produce antibacterial substances [48]. Interestingly, only strain F2 showed antibacterial activity against the gram-negative bacterium *E. coli*. *B. amyloliquefaciens* F2 demonstrated the broadest and most potent antibacterial activity, effectively inhibiting *E. coli*, *S. aureus*, *L. monocytogenes*, and *P. aeruginosa*. This result aligns with those of previous studies, which indicated that *B. amyloliquefaciens* generally exhibit superior antibacterial activity against pathogens compared to other *Bacillus* species [49, 50].

### Microbial Safety Analysis of *Bacillus* spp.

The results of the microbial safety assessment of the eight *Bacillus* isolates are presented in Table 3. Hemolysis refers to the destruction of red blood cells by substances produced by microorganisms that can lead to abnormal red blood cell function [51]. All eight *Bacillus* isolates were identified as γ-type (data non-shown), indicating no hemolytic activity [52].

Analysis of the biogenic amine productivity of *Bacillus* spp. showed that F3 and F7 produced putrescine, whereas S1 produced cadaverine and tyramine (Table 3). S3 produced putrescine, cadaverine, and tyramine. In contrast, the strains F2, S2, S4, and A1 did not produce biogenic amines. Moon *et al*. (2015) reported that *B. subtilis* and *B. licheniformis* isolated from *Cheonggukjang*, a Korean fermented food, do not produce biogenic amines [38]. Lee *et al*. (2017) also reported that three strains of *B. amyloliquefaciens* isolated from soy sauce did not produce biogenic amines [53]. Biogenic amines can be found in fermented foods such as dairy and soybean products and can be harmful to humans when consumed in large amounts [54]. Therefore, the strains F2, F2, S4, and A1, which do not produce biogenic amines, not only help maintain the quality of fermented foods, but also exhibit low pathogenicity risk, and can be easily applied to commercial fermentation processes.

### Protein Extraction of *C. pyrenoidosa* for Utilizing Food Materials

Table 4 presents the protein extraction yields from *C. pyrenoidosa* using the eight *Bacillus* spp. as fermentation starters. Strain F2 showed the highest protein extraction yield of 34.45 ± 1.21%, and F3 showed the second highest yield of 21.96±0.78% (*p* < 0.05). Next, S2 showed 16.46 ± 1.02%; S4, 13.93±0.6%; F7, 13.67±0.7%; S1, 7.78±0.29%; S3, 6.99 ± 1.24%; and A1, 5.66 ± 0.01%, in that order. The high protein extraction yields for F2 and F3 are attributed to their superior cellulase and protease activities (Fig. 2). Protein extraction yields from *C. pyrenoidosa* fermentation by three strains of *B. subtilis* (S1, S2, and S3) ranged from 6.99 ± 1.24% to 16.46 ± 1.02%. These differences were attributed to variations in enzyme activity, despite all the strains belonging to the same species. The *C. pyrenoidosa* cell wall is composed of polysaccharides and glycoproteins [55], making it susceptible to decomposition by extracellular enzymes, such as cellulase and proteases, produced during fermentation. This is supported by results indicating that strain F2, which possessed the highest cellulase, protease, and lipase activities, effectively extracted proteins from *C. pyrenoidosa*. In addition, our previous study confirmed that the protein extraction yield from *C. pyrenoidosa* was approximately 35% when using proteases derived from *Bacillus* spp. [55]. This result is similar to the *C. pyrenoidosa* protein extraction yield using strain F2, suggesting that microbial fermentation can effectively decompose *C. pyrenoidosa* cell walls. Safi *et al*. (2014) reported that the protein extraction yield of *Chlorella vulgaris* was 33.20% when alkaline treatment was performed for 2 h at pH 12 [56], and Wang and Jang (2012) reported that the protein extraction yield of *C. pyrenoidosa* was 22.90% after freeze-thawed and sonicated [57]. This suggests that *Bacillus* spp. treatment is an efficient method for *C. pyrenoidosa* protein extraction. Consequently, microbial fermentation, such as *B. amyloliquefaciens* F2, has the potential to expand the commercial utilization of marine protein sources, such as *C. pyrenoidosa*. In future, it will be necessary to study the optimal enzyme activity conditions for *B. amyloliquefaciens* F2 protein properties and scale-up to provide a basic technology for alternative protein industrial applications.

## Conclusion

In conclusion, we successfully isolated and characterized a total of twenty-two *Bacillus* spp. from several *Jeotgal* samples that are considered to have significant biotechnological potential. isolated *Bacillus* spp. from flounder, squid, and anchovy *Jeotgal* were evaluated for their roles in fermentation. These *Bacillus* isolates were functionally identified to have effective protease, lipase, and cellulase activities. These can be employed as agents to enhance biomass degradation necessary to produce bioactive compounds and improve food processing. The antibacterial properties of isolated *Bacillus* strains have been recognized as potential antimicrobial agents that can control the growth of microbial pathogens, thereby ensuring the safety of food during fermentation. In particular, F2, S2, S4, and A1, which do not produce biogenic amines, were confirmed to be safe for commercial fermentation applications. Additionally, this study demonstrates that using *Bacillus* spp. in fermentation improves protein extraction yields from *C. pyrenoidosa* and indicates the effectiveness of microbial fermentation with *Bacillus* strains in improving protein extraction from microalgae. Notably, *B. amyloliquefaciens* F2, isolated in this study, exhibited a *C. pyrenoidosa* protein extraction yield of 34.45 ± 1.21%, highlighting its potential to enhance conventional microalgal protein extraction methods. Finally, this study supports the commercial viability of using *Bacillus* spp. to extract proteins from marine biomass. Future studies should optimize the growth conditions and enzyme production, particularly for *B. amyloliquefaciens* F2. Furthermore, it is essential to explore the scaling up of protein extraction from microalgal species, such as *C. pyrenoidosa*, for commercial applications

## Supplemental Materials

Supplementary data for this paper are available on-line only at http://jmb.or.kr.



## Figures and Tables

**Table 1:** Identification of *Bacillus* spp. from three types of *Jeotgal* based on 16S rRNA gene sequence.

Source	Strain No.	Identification by 16S rRNA gene sequence		
		Identified strains	Similarity (%)	GenBank Accesion NO.
*Flounder* *Jeotgal*	F1	*Bacillus altitudinis* AC11.2	100.00	KY416926.1
	F2	*Bacillus amyloliquefaciens* F2	99.80	OQ561751.1
	F3	*Bacillus vallismortis* SAB12	100.00	OQ566834.1
	F4	*Bacillus subtilis* SRCM125727	100.00	CP116773.1
	F5	*Bacillus licheniformis* CARE_V8	100.00	MN865990.1
	F6	*Bacillus safensis* SAB8	100.00	OQ566830.1
	F7	*Bacillus velezensis* ZY1	100.00	CP117945.1
	F8	*Bacillus amyloliquefaciens* 802	100.00	MT585518.1
	F9	*Bacillus paralicheniformis* SAB7	100.00	OQ566829.1
	F10	*Bacillus atrophaeus* DE-2	100.00	MT240913.1
	F11	*Bacillus mojavensis* F11	99.93	MT043920.1
*Squid Jeotga1*	S1	*Bacillus subtilis* MMS	100.00	OQ553759.1
	S2	*Bacillus subtilis* HY-88	100.00	MZ895433.1
	S3	*Bacillus subtilis* ZIM3	100.00	MK250654.1
	S4	*Bacillus rugosus* SPB7	100.00	OR740574.1
	S5	*Bacillus* sp. (in: Bacteria) S5	99.86	MK656945.1
	S6	*Bacillus subtilis* PEBSB307040301	100.00	FJ685766.1
	S7	*Bacillus subtilis* Md1-42	100.00	MF581448.1
	S8	*Bacillus megaterium* AK4	100.00	OQ875860.1
*Anchovy* *Jeotgal*	A1	*Bacillus altitudinis* LNPC8	100.00	OM319769.1
	A2	*Bacillus safensis* A2	99.80	MZ674177.1
	A3	*Bacillus australimaris* ROA027	100.00	OP890644.1

**Table 2:** Antibacterial activity of the *Bacillus* spp. from *Jeotgal* against five types of pathogenic bacteria (*Escherichia coli*, *Staphylococcus aureus*, *Listeria monocytogenes*, *Bacillus cereus*, *Pseudomonas aeruginosa*).

Strain No.	*Escherichia coli*	*Staphylococcus aureus*	*Listeria monocytogenes*	*Bacillus cereus*	*Pseudomonas aeruginosa*
F2	1.70 ± 0.02*	1.48 ± 0.05	2.30 ± 0.02	-	1.41 ± 0.02
F3	-	-	1.40 ± 0.05	-	-
F7	-	1.20 ± 0.02	1.30 ± 0.02	-	-
S1	-	-	-	-	-
S2	-	-	1.50 ± 0.05	-	-
S3	-	-	1.10 ± 0.00	-	-
S4	-	1.20 ± 0.00	1.80 ± 0.03	-	-
A1	-	-	1.30 ± 0.01	-	-

*Antibacterial activity (ratio) = clearance zone size (mm) / colony size (mm); -, means no antibacterial activity.

**Table 3:** Microbial safety evaluation of the isolated *Bacillus* spp. strains.

Strain No.	Biogenic amine productivity			
	Cadaverine	Putrescine	Tyramine	Histamine
F2	-*	-	-	-
F3	-	+	-	-
F7	-	+	-	-
S1	+	-	+	-
S2	-	-	-	-
S3	+	+	+	-
S4	-	-	-	-
A1	-	-	-	-

*-, means negative in biogenic amine productivity; +, means positive in biogenic amine productivity.

**Table 4:** Protein extraction yield from *Chlorella pyrenoidosa* by fermentation with *Bacillus* spp. isolated from *Jeotgal*.

Strain No.	Yield of protein extraction (%)
Control*	4.10 ± 0.44^g^
F2	34.45 ± 1.21^a^
F3	21.96 ± 0.78^b^
F7	13.67 ± 0.7^d^
S1	7.78 ± 0.29^e^
S2	16.46 ± 1.02^c^
S3	6.99 ± 1.24^ef^
S4	13.93 ± 0.6^d^
A1	5.66 ± 0.01^f^

*Control, *C. pyrenoidosa* medium cultured at 37°C for 24 h without uninoculated *Bacillus* spp.. Values with different letters vary significantly (*p* < 0.05).
