# Supplementary material for: Protein Extraction from Chlorella pyrenoidosa Using Bacillus spp. Isolated from Jeotgal: Strain isolation, Characterization, and Fermentation
Source: J Microbiol Biotechnol. 2025 May 15;35:e2411070. doi: 10.4014/jmb.2411.11070 (PMC12099625; doi:10.4014/jmb.2411.11070)

J. Microbiol. Biotechnol. 2025. 35: e2411070
https://doi.org/10.4014/jmb.2411.11070

Supplementary Tables and Figure

Protein Extraction from *Chlorella pyrenoidosa* Using *Bacillus* spp. Isolated from *Jeotgal*: Strain isolation, Characterization, and Fermentation

Kyung-Jin Cho[1,2], Min-Ung Kim[1], Geum-Jae Jeong[1,2], Do Kyung Oh[2], Ju-Hong Kang[1], Da-Hyeon Yoon[1], Fazlurrahman Khan[2,3,4], and Young-Mog Kim[1,2*]

[1]*Department of Food Science and Technology, Pukyong National University, Busan 48513, Republic of Korea*
[2]*Research Center for Marine Integrated Bionics Technology, Pukyong National University, Busan 48513, Republic of Korea*
[3]*Ocean and Fisheries Development International Cooperation Institute, Pukyong National University, Busan 48513, Republic of Korea*
[4]*International Graduate Program of Fisheries Science, Pukyong National University, Busan 48513, Republic of Korea*

*Corresponding author: ymkim@pknu.ac.kr (Y.M. Kim)

Supplementary Table S1. The full sequencing results of *Bacillus* spp. isolated from *Jeotgal*

Strain No.	Full sequencing results
F1	TCGAGCGGACAGAAGGGAGCTTGCTCCCGGATGTTAGCGGCGGACGG<br>GTGAGTAACACGTGGGTAACCTGCCTGTAAGACTGGGATAACTCCGG<br>GAAACCGGAGCTAATACCGGATAGTTCCTTGAACCGCATGGTTCAAG<br>GATGAAAGACGGTTTCGGCTGTCACTTACAGATGGACCCGCGGCGCA<br>TTAGCTAGTTGGTGAGGTAACGGCTCACCAAGGCGACGATGCGTAGC<br>CGACCTGAGAGGGTGATCGGCCACACTGGGACTGAGACACGGCCCAG<br>ACTCCTACGGGAGGCAGCAGTAGGGAATCTTCCGCAATGGACGAAAG<br>TCTGACGGAGCAACGCCGCGTGAGTGATGAAGGTTTTCGGATCGTAA<br>AGCTCTGTTGTTAGGGAAGAACAAGTGCAAGAGTAACTGCTTGCACC<br>TTGACGGTACCTAACCAGAAAGCCACGGCTAACTACGTGCCAGCAGC<br>CGCGGTAATACGTAGGTGGCAAGCGTTGTCCGGAATTATTGGGCGTA<br>AAGGGCTCGCAGGCGGTTTCTTAAGTCTGATGTGAAAGCCCCCGGCTC<br>AACCGGGGAGGGTCATTGGAAACTGGGAAACTTGAGTGCAGAAGAG<br>GAGAGTGGAATTCCACGTGTAGCGGTGAAATGCGTAGAGATGTGGAG<br>GAACACCAGTGGCGAAGGCGACTCTCTGGTCTGTAACTGACGCTGAG<br>GAGCGAAAGCGTGGGGAGCGAACAGGATTAGATACCCTGGTAGTCCA<br>CGCCGTAAACGATGAGTGCTAAGTGTTAGGGGGTTTCCGCCCCTTAGT<br>GCTGCAGCTAACGCATTAAGCACTCCGCCTGGGGAGTACGGTCGCAA<br>GACTGAAACTCAAAGGAATTGACGGGGGCCCGCACAAGCGGTGGAG<br>CATGTGGTTTAATTCGAAGCAACGCGAAGAACCTTACCAGGTCTTGAC<br>ATCCTCTGACAACCCTAGAGATAGGGCTTTCCCTTCGGGGACAGAGTG<br>ACAGGTGGTGCATGGTTGTCGTCAGCTCGTGTCGTGAGATGTTGGGTT<br>AAGTCCCGCAACGAGCGCAACCCTTGATCTTAGTTGCCAGCATTCAGT<br>TGGGCACTCTAAGGTGACTGCCGGTGACAAACCGGAGGAAGGTGGGG<br>ATGACGTCAAATCATCATGCCCCTTATGACCTGGGCTACACACGTGCT<br>ACAATGGACAGAACAAAGGGCTGCGAGACCGCAAGGTTTAGCCAATC<br>CCACAAATCTGTTCTCAGTTCGGATCGCAGTCTGCAACTCGACTGCGT<br>GAAGCTGGAATCGCTAGTAATCGCGGATCAGCATGCCGCGGTGAATA<br>CGTTCCCGGGCCTTGTACACACCGCCCGTCACACCACGAGAGTTTGCA<br>ACACCCGAAGTCGGTGAGGTAACC
F2	AGCAAGGCCGCGTTCCCTAATACATGCAAGTCGAGCGGACAGATGGG<br>AGCTTGCTCCCTGATGTTAGCGGCGGACGGGTGAGTAACACGTGGGT<br>AACCTGCCTGTAAGACTGGGATAACTCCGGGAAACCGGGGCTAATAC<br>CGGATGGTTGTCTGAACCGCATGGTTCAGACATAAAAGGTGGCTTCG<br>GCTACCACTTACAGATGGACCCGCGGCGCATTAGCTAGTTGGTGAGG<br>TAACGGCTCACCAAGGCGACGATGCGTAGCCGACCTGAGAGGGTGAT<br>CGGCCACACTGGGACTGAGACACGGCCCAGACTCCTACGGGAGGCAG<br>CAGTAGGGAATCTTCCGCAATGGACGAAAGTCTGACGGAGCAACGCC<br>GCGTGAGTGATGAAGGTTTTCGGATCGTAAAGCTCTGTTGTTAGGGAA<br>GAACAAGTGCCGTTCAAATAGGGCGGCACCTTGACGGTACCTAACCA<br>GAAAGCCACGGCTAACTACGTGCCAGCAGCCGCGGTAATACGTAGGT<br>GGCAAGCGTTGTCCGGAATTATTGGGCGTAAAGGGCTCGCAGGCGGT<br>TTCTTAAGTCTGATGTGAAAGCCCCCGGCTCAACCGGGGAGGGTCATT

	GGAAACTGGGGAACTTGAGTGCAGAAGAGGAGAGTGGAATTCCACGTGTAGCGGTGAAATGCGTAGAGATGTGGAGGAACACCAGTGGCGAAGGCGACTCTCTGGTCTGTAACTGACGCTGAGGAGCGAAAGCGTGGGGAGCGAACAGGATTAGATACCCTGGTAGTCCACGCCGTAAACGATGAGTGCTAAGTGTTAGGGGGTTTCCGCCCCTTAGTGCTGCAGCTAACGCATTAAGCACTCCGCCTGGGGAGTACGGTCGCAAGACTGAAACTCAAAGGAATTGACGGGGGCCCGCACAAGCGGTGGAGCATGTGGTTTAATTCGAAGCAACGCGAAGAACCTTACCAGGTCTTGACATCCTCTGACAATCCTAGAGATAGGACGTCCCCTTCGGGGGCAGAGTGACAGGTGGTGCATGGTTGTCGTCAGCTCGTGTCGTGAGATGTTGGGTTAAGTCCCGCAACGAGCGCAACCCTTGATCTTAGTTGCCAGCATTCAGTTGGGCACTCTAAGGTGACTGCCGGTGACAAACCGGAGGAAGGTGGGGATGACGTCAAATCATCATGCCCCTTATGACCTGGGCTACACACGTGCTACAATGGACAGAACAAAGGGCAGCGAAACCGCGAGGTTAAGCCAATCCCACAAATCTGTTCTCAGTTCGGATCGCAGTCTGCAACTCGACTGCGTGAAGCTGGAATCGCTAGTAATCGCGGATCAGCATGCCGCGGTGAATACGTTCCCGGGCCTTGTACACACCGCCCGTCACACCACGAGAGTTTGTAACACCCGAAGTCGGTG
F3	GCGTAGCCGACCTGAGAGGGTGATCGGCCACACTGGGACTGAGACACGGCCCAGACTCCTACGGGAGGCAGCAGTAGGGAATCTTCCGCAATGGACGAAAGTCTGACGGAGCAACGCCGCGTGAGTGATGAAGGTTTTCGGATCGTAAAGCTCTGTTGTTAGGGAAGAACAAGTGCCGTTCAAATAGGGCGGCACCTTGACGGTACCTAACCAGAAAGCCACGGCTAACTACGTGCCAGCAGCCGCGGTAATACGTAGGTGGCAAGCGTTGTCCGGAATTATTGGGCGTAAAGGGCTCGCAGGCGGTTTCTTAAGTCTGATGTGAAAGCCCCCGGCTCAACCGGGGAGGGTCATTGGAAACTGGGGAACTTGAGTGCAGAAGAGGAGAGTGGAATTCCACGTGTAGCGGTGAAATGCGTAGAGATGTGGAGGAACACCAGTGGCGAAGGCGACTCTCTGGTCTGTAACTGACGCTGAGGAGCGAAAGCGTGGGGAGCGAACAGGATTAGATACCCTGGTAGTCCACGCCGTAAACGATGAGTGCTAAGTGTTAGGGGGTTTCCGCCCCTTAGTGCTGCAGCTAACGCATTAAGCACTCCGCCTGGGGAGTACGGTCGCAAGACTGAAACTCAAAGGAATTGACGGGGGCCCGCACAAGCGGTGGAGCATGTGGTTTAATTCGAAGCAACGCGAAGAACCTTACCAGGTCTTGACATCCTCTGACAATCCTAGAGATAGGACGTCCCCTTCGGGGGCAGAGTGACAGGTGGTGCATGGTTGTCGTCAGCTCGTGTCGTGAGATGTTGGGTTAAGTCCCGCAACGAGCGCAACCCTTGATCTTAGTTGCCAGCATTCAGTTGGGCACTCTAAGGTGACTGCCGGTGACAAACCGGAGGAAGGTGGGGATGACGTCAAATCATCATGCCCCTTATGACCTGGGCTACACACGTGCTACAATGGACAGAACAAAGGGCAGCGAAACCGCGAGGTTAAGCCAATCCCACAAATCTGTTCTCAGTTCGGATCGCAGTCTGCAACTCGACTGCGTGAAGCTGGAATCGCTAGTAATCGCGGATCAGCATGCCGCGGTGAATACGTTCCCGGGCCTTGTACACACCGCCCGTCACACCACGAGAGTTTGTAACACCCGAAGTCGGTGAGG
F4	GTAACACGTGGGTAACCTGCCTGTAAGACTGGGATAACTCCGGGAAACCGGGGCTAATACCGGATGGTTGTTTGAACCGCATGGTTCAAACATAAAAGGTGGCTTCGGCTACCACTTACAGATGGACCCGCGGCGCATTAGCTAGTTGGTGAGGTAACGGCTCACCAAGGCAACGATGCGTAGCCGACCTGAGAGGGTGATCGGCCACACTGGGACTGAGACACGGCCCAGACTCCTACGGGAGGCAGCAGTAGGGAATCTTCCGCAATGGACGAAAGTCTGACGGAGCAACGCCGCGTGAGTGATGAAGGTTTTCGGATCGTAAAGCT

	CTGTTGTTAGGGAAGAACAAGTACCGTTCGAATAGGGCGGTACCTTG<br>ACGGTACCTAACCAGAAAGCCACGGCTAACTACGTGCCAGCAGCCGC<br>GGTAATACGTAGGTGGCAAGCGTTGTCCGGAATTATTGGGCGTAAAG<br>GGCTCGCAGGCGGTTTCTTAAGTCTGATGTGAAAGCCCCCGGCTCAAC<br>CGGGGAGGGTCATTGGAAACTGGGGAACTTGAGTGCAGAAGAGGAG<br>AGTGGAATTCCACGTGTAGCGGTGAAATGCGTAGAGATGTGGAGGAA<br>CACCAGTGGCGAAGGCGACTCTCTGGTCTGTAACTGACGCTGAGGAG<br>CGAAAGCGTGGGGAGCGAACAGGATTAGATACCCTGGTAGTCCACGC<br>CGTAAACGATGAGTGCTAAGTGTTAGGGGGTTTCCGCCCCTTAGTGCT<br>GCAGCTAACGCATTAAGCACTCCGCCTGGGGAGTACGGTCGCAAGAC<br>TGAAACTCAAAGGAATTGACGGGGGCCCGCACAAGCGGTGGAGCATG<br>TGGTTTAATTCGAAGCAACGCGAAGAACCTTACCAGGTCTTGACATCC<br>TCTGACAATCCTAGAGATAGGACGTCCCCTTCGGGGGCAGAGTGACA<br>GGTGGTGCATGGTTGTCGTCAGCTCGTGTCGTGAGATGTTGGGTTAAG<br>TCCCGCAACGAGCGCAACCCTTGATCTTAGTTGCCAGCATTCAGTTGG<br>GCACTCTAAGGTGACTGCCGGTGACAAACCGGAGGAAGGTGGGGATG<br>ACGTCAAATCATCATGCCCCTTATGACCTGGGCTACACACGTGCTACA<br>ATGGACAGAACAAAGGGCAGCGAAACCGCGAGGTTAAGCCAATCCC<br>ACAAATCTGTTCTCAGTTCGGATCGCAGTCTGCAACTCGACTGCGTGA<br>AGCTGGAATCGCTAGTAATCGCGGATCAGCATGCCGCGGTG
F5	CCTGATGTCAGCGGCGGACGGGTGAGTAACACGTGGGTAACCTGCCT<br>GTAAGACTGGGATAACTCCGGGAAACCGGGGCTAATACCGGATGCTT<br>GATTGAACCGCATGGTTCAATTATAAAAGGTGGCTTTTAGCTACCACT<br>TACAGATGGACCCGCGGCGCATTAGCTAGTTGGTGAGGTAACGGCTC<br>ACCAAGGCAACGATGCGTAGCCGACCTGAGAGGGTGATCGGCCACAC<br>TGGGACTGAGACACGGCCCAGACTCCTACGGGAGGCAGCAGTAGGGA<br>ATCTTCCGCAATGGACGAAAGTCTGACGGAGCAACGCCGCGTGAGTG<br>ATGAAGGTTTTCGGATCGTAAAACTCTGTTGTTAGGGAAGAACAAGT<br>ACCGTTCGAATAGGGCGGTACCTTGACGGTACCTAACCAGAAAGCCA<br>CGGCTAACTACGTGCCAGCAGCCGCGGTAATACGTAGGTGGCAAGCG<br>TTGTCCGGAATTATTGGGCGTAAAGCGCGCGCAGGCGGTTTCTTAAGT<br>CTGATGTGAAAGCCCCCGGCTCAACCGGGGAGGGTCATTGGAAACTG<br>GGGAACTTGAGTGCAGAAGAGGAGAGTGGAATTCCACGTGTAGCGGT<br>GAAATGCGTAGAGATGTGGAGGAACACCAGTGGCGAAGGCGACTCTC<br>TGGTCTGTAACTGACGCTGAGGCGCGAAAGCGTGGGGAGCGAACAGG<br>ATTAGATACCCTGGTAGTCCACGCCGTAAACGATGAGTGCTAAGTGTT<br>AGAGGGTTTCCGCCCTTTAGTGCTGCAGCAAACGCATTAAGCACTCCG<br>CCTGGGGAGTACGGTCGCAAGACTGAAACTCAAAGGAATTGACGGGG<br>GCCCGCACAAGCGGTGGAGCATGTGGTTTAATTCGAAGCAACGCGAA<br>GAACCTTACCAGGTCTTGACATCCTCTGACAACCCTAGAGATAGGGCT<br>TCCCCTTCGGGGGCAGAGTGACAGGTGGTGCATGGTTGTCGTCAGCTC<br>GTGTCGTGAGATGTTGGGTTAAGTCCCGCAACGAGCGCAACCCTTGAT<br>CTTAGTTGCCAGCATTCAGTTGGGCACTCTAAGGTGACTGCCGGTGAC<br>AAACCGGAGGAAGGTGGGGATGACGTCAAATCATCATGCCCCTTATG<br>ACCTGGGCTACACACGTGCTACAATGGGCAGAACAAAGGGCAGCGAA<br>GCCGCGAGGCTAAGCCAATCCCACAAATCTGTTCTCAGTTCGGATCGC<br>AGTCTGCAACTCGACTGCGTGAAGCTGGAATCGCTAGTAATCGCGGA<br>TCAGCATGCCGCGGTGAATACGTTCCCGGGCCTTGTACACACCGCCCG<br>TCACACCACGAGAGTTTGTAACACCCGAAGTCGGTGA

F6	GTGAGTAACACGTGGGTAACCTGCCTGTAAGACTGGGATAACTCCGGGAAACCGGAGCTAATACCGGATAGTTCCTTGAACCGCATGGTTCAAGGATGAAAGACGGTTTCGGCTGTCACTTACAGATGGACCCGCGGCGCATTAGCTAGTTGGTGGGGTAATGGCTCACCAAGGCGACGATGCGTAGCCGACCTGAGAGGGTGATCGGCCACACTGGGACTGAGACACGGCCCAGACTCCTACGGGAGGCAGCAGTAGGGAATCTTCCGCAATGGACGAAAGTCTGACGGAGCAACGCCGCGTGAGTGATGAAGGTTTTCGGATCGTAAAGCTCTGTTGTTAGGGAAGAACAAGTGCGAGAGTAACTGCTCGCACCTTGACGGTACCTAACCAGAAAGCCACGGCTAACTACGTGCCAGCAGCCGCGGTAATACGTAGGTGGCAAGCGTTGTCCGGAATTATTGGGCGTAAAGGGCTCGCAGGCGGTTTCTTAAGTCTGATGTGAAAGCCCCCGGCTCAACCGGGGAGGGTCATTGGAAACTGGGAAACTTGAGTGCAGAAGAGGAGAGTGGAATTCCACGTGTAGCGGTGAAATGCGTAGAGATGTGGAGGAACACCAGTGGCGAAGGCGACTCTCTGGTCTGTAACTGACGCTGAGGAGCGAAAGCGTGGGGAGCGAACAGGATTAGATACCCTGGTAGTCCACGCCGTAAACGATGAGTGCTAAGTGTTAGGGGGTTTCCGCCCCTTAGTGCTGCAGCTAACGCATTAAGCACTCCGCCTGGGGAGTACGGTCGCAAGACTGAAACTCAAAGGAATTGACGGGGGCCCGCACAAGCGGTGGAGCATGTGGTTTAATTCGAAGCAACGCGAAGAACCTTACCAGGTCTTGACATCCTCTGACAACCCTAGAGATAGGGCTTTCCCTTCGGGGACAGAGTGACAGGTGGTGCATGGTTGTCGTCAGCTCGTGTCGTGAGATGTTGGGTTAAGTCCCGCAACGAGCGCAACCCTTGATCTTAGTTGCCAGCATTCAGTTGGGCACTCTAAGGTGACTGCCGGTGACAAACCGGAGGAAGGTGGGGATGACGTCAAATCATCATGCCCCTTATGACCTGGGCTACACACGTGCTACAATGGACAGAACAAAGGGCTGCAAGACCGCAAGGTTTAGCCAATCCCATAAATCTGTTCTCAGTTCGGATCGCAGTCTGCAACTCGACTGCGTGAAGCTGGAATCGCTAGTAATCGCGGATCAGCATGCCGCGGTGAATACGTTCCCGGGCCTTGTACACACCGCCCGTCACACCACGAGAGTTTGCAACACCCGAAGTCGGTG
F7	GGACAGATGGGAGCTTGCTCCCTGATGTTAGCGGCGGACGGGTGAGTAACACGTGGGTAACCTGCCTGTAAGACTGGGATAACTCCGGGAAACCGGGGCTAATACCGGATGGTTGTTTGAACCGCATGGTTCAGACATAAAAGGTGGCTTCGGCTACCACTTACAGATGGACCCGCGGCGCATTAGCTAGTTGGTGAGGTAACGGCTCACCAAGGCGACGATGCGTAGCCGACCTGAGAGGGTGATCGGCCACACTGGGACTGAGACACGGCCCAGACTCCTACGGGAGGCAGCAGTAGGGAATCTTCCGCAATGGACGAAAGTCTGACGGAGCAACGCCGCGTGAGTGATGAAGGTTTTCGGATCGTAAAGCTCTGTTGTTAGGGAAGAACAAGTGCCGTTCAAATAGGGCGGCACCTTGACGGTACCTAACCAGAAAGCCACGGCTAACTACGTGCCAGCAGCCGCGGTAATACGTAGGTGGCAAGCGTTGTCCGGAATTATTGGGCGTAAAGGGCTCGCAGGCGGTTTCTTAAGTCTGATGTGAAAGCCCCCGGCTCAACCGGGGAGGGTCATTGGAAACTGGGGAACTTGAGTGCAGAAGAGGAGAGTGGAATTCCACGTGTAGCGGTGAAATGCGTAGAGATGTGGAGGAACACCAGTGGCGAAGGCGACTCTCTGGTCTGTAACTGACGCTGAGGAGCGAAAGCGTGGGGAGCGAACAGGATTAGATACCCTGGTAGTCCACGCCGTAAACGATGAGTGCTAAGTGTTAGGGGGTTTCCGCCCCTTAGTGCTGCAGCTAACGCATTAAGCACTCCGCCTGGGGAGTACGGTCGCAAGACTGAAACTCAAAGGAATTGACGGGGGCCCGCACAAGCGGTGGAGCATGTGGTTTAATTCGAAGCAACGCGAAGAACCTTACCAGGTCTTGACATCCTCTGACAATCCTAGAGATAGGACGTCCCCTTCGGGGGCAGAGTGACAGG

	TGGTGCATGGTTGTCGTCAGCTCGTGTCGTGAGATGTTGGGTTAAGTC<br>CCGCAACGAGCGCAACCCTTGATCTTAGTTGCCAGCATTCAGTTGGGC<br>ACTCTAAGGTGACTGCCGGTGACAAACCGGAGGAAGGTGGGGATGAC<br>GTCAAATCATCATGCCCCTTATGACCTGGGCTACACACGTGCTACAAT<br>GGACAGAACAAAGGGCAGCGAAACCGCGAGGTTAAGCCAATCCCAC<br>AAATCTGTTCTCAGTTCGGATCGCAGTCTGCAACTCGACTGCGTGAAG<br>CTGGAATCGCTAGTAATCGCGGATCAGCATGCCGCGGTGAATACGTT<br>CCCGGGCCTTGTACACACCGCCCGTCACACCA
F8	CGGGTGAGTAACACGTGGGTAACCTGCCTGTAAGACTGGGATAACTC<br>CGGGAAACCGGGGCTAATACCGGATGGTTGTTTGAACCGCATGGTTC<br>AGACATAAAAGGTGGCTTCGGCTACCACTTACAGATGGACCCGCGGC<br>GCATTAGCTAGTTGGTGAGGTAACGGCTCACCAAGGCGACGATGCGT<br>AGCCGACCTGAGAGGGTGATCGGCCACACTGGGACTGAGACACGGCC<br>CAGACTCCTACGGGAGGCAGCAGTAGGGAATCTTCCGCAATGGACGA<br>AAGTCTGACGGAGCAACGCCGCGTGAGTGATGAAGGTTTTCGGATCG<br>TAAAGCTCTGTTGTTAGGGAAGAACAAGTGCCGTTCAAATAGGGCGG<br>CACCTTGACGGTACCTAACCAGAAAGCCACGGCTAACTACGTGCCAG<br>CAGCCGCGGTAATACGTAGGTGGCAAGCGTTGTCCGGAATTATTGGG<br>CGTAAAGGGCTCGCAGGCGGTTTCTTAAGTCTGATGTGAAAGCCCCC<br>GGCTCAACCGGGGAGGGTCATTGGAAACTGGGGAACTTGAGTGCAGA<br>AGAGGAGAGTGGAATTCCACGTGTAGCGGTGAAATGCGTAGAGATGT<br>GGAGGAACACCAGTGGCGAAGGCGACTCTCTGGTCTGTAACTGACGC<br>TGAGGAGCGAAAGCGTGGGGAGCGAACAGGATTAGATACCCTGGTA<br>GTCCACGCCGTAAACGATGAGTGCTAAGTGTTAGGGGGTTTCCGCCCC<br>TTAGTGCTGCAGCTAACGCATTAAGCACTCCGCCTGGGGAGTACGGTC<br>GCAAGACTGAAACTCAAAGGAATTGACGGGGGCCCGCACAAGCGGT<br>GGAGCATGTGGTTTAATTCGAAGCAACGCGAAGAACCTTACCAGGTC<br>TTGACATCCTCTGACAATCCTAGAGATAGGACGTCCCCTTCGGGGGCA<br>GAGTGACAGGTGGTGCATGGTTGTCGTCAGCTCGTGTCGTGAGATGTT<br>GGGTTAAGTCCCGCAACGAGCGCAACCCTTGATCTTAGTTGCCAGCAT<br>TCAGTTGGGCACTCTAAGGTGACTGCCGGTGACAAACCGGAGGAAGG<br>TGGGGATGACGTCAAATCATCATGCCCCTTATGACCTGGGCTACACAC<br>GTGCTACAATGGACAGAACAAAGGGCAGCGAAACCGCGAGGTTAAG<br>CCAATCCCACAAATCTGTTCTCAGTTCGGATCGCAGTCTGCAACTCGA<br>CTGCGTGAAGCTGGAATCGCTAGTAATCGCGGATCAGCATGCCGCGG<br>TGAATACGTTCCCGGGCCTTGTACACACCGCCCGTCACACCACGAGA<br>GTTTGTAACACCCGAAGTCGGTGAGGTAACCTTTATGGAGC
F9	ACGTGGGTAACCTGCCTGTAAGACTGGGATAACTCCGGGAAACCGGG<br>GCTAATACCGGATGCTTGATTGAACCGCATGGTTCAATTATAAAAGGT<br>GGCTTTTAGCTACCACTTACAGATGGACCCGCGGCGCATTAGCTAGTT<br>GGTGAGGTAACGGCTCACCAAGGCAACGATGCGTAGCCGACCTGAGA<br>GGGTGATCGGCCACACTGGGACTGAGACACGGCCCAGACTCCTACGG<br>GAGGCAGCAGTAGGGAATCTTCCGCAATGGACGAAAGTCTGACGGAG<br>CAACGCCGCGTGAGTGATGAAGGTTTTCGGATCGTAAAACTCTGTTGT<br>TAGGGAAGAACAAGTACCGTTCGAATAGGGCGGTACCTTGACGGTAC<br>CTAACCAGAAAGCCACGGCTAACTACGTGCCAGCAGCCGCGGTAATA<br>CGTAGGTGGCAAGCGTTGTCCGGAATTATTGGGCGTAAAGCGCGCGC<br>AGGCGGTTTCTTAAGTCTGATGTGAAAGCCCCCGGCTCAACCGGGGA<br>GGGTCATTGGAAACTGGGGAACTTGAGTGCAGAAGAGGAGAGTGGA<br>ATTCCACGTGTAGCGGTGAAATGCGTAGAGATGTGGAGGAACACCAG

	TGGCGAAGGCGACTCTCTGGTCTGTAACTGACGCTGAGGCGCGAAAG<br>CGTGGGGAGCGAACAGGATTAGATACCCTGGTAGTCCACGCCGTAAA<br>CGATGAGTGCTAAGTGTTAGAGGGTTTCCGCCCTTTAGTGCTGCAGCA<br>AACGCATTAAGCACTCCGCCTGGGGAGTACGGTCGCAAGACTGAAAC<br>TCAAAGGAATTGACGGGGGCCCGCACAAGCGGTGGAGCATGTGGTTT<br>AATTCGAAGCAACGCGAAGAACCTTACCAGGTCTTGACATCCTCTGA<br>CAACCCTAGAGATAGGGCTTCCCCTTCGGGGGCAGAGTGACAGGTGG<br>TGCATGGTTGTCGTCAGCTCGTGTCGTGAGATGTTGGGTTAAGTCCCG<br>CAACGAGCGCAACCCTTGATCTTAGTTGCCAGCATTCAGTTGGGCACT<br>CTAAGGTGACTGCCGGTGACAAACCGGAGGAAGGTGGGGATGACGTC<br>AAATCATCATGCCCCTTATGACCTGGGCTACACACGTGCTACAATGGG<br>CAGAACAAAGGGCAGCGAAGCCGCGAGGCTAAGCCAATCCCACAAA<br>TCTGTTCTCAGTTCGGATCGCAGTCTGCAACTCGACTGCGTGAAGCTG<br>GAATCGCTAGTAATCGCGGATCAGCATGCCGCGGTGAATACGTTCCC<br>GGGCCTTGTACACACCGCCCGTCACACCACGAGAGTTTGTAACACCC<br>GAAGT
F10	AAACCGGGGCTAATACCGGATGCTTGTTTGAACCGCATGGTTCAAAC<br>ATAAAAGGTGGCTTCGGCTACCACTTACAGATGGACCCGCGGCGCAT<br>TAGCTAGTTGGTGAGGTAACGGCTCACCAAGGCAACGATGCGTAGCC<br>GACCTGAGAGGGTGATCGGCCACACTGGGACTGAGACACGGCCCAGA<br>CTCCTACGGGAGGCAGCAGTAGGGAATCTTCCGCAATGGACGAAAGT<br>CTGACGGAGCAACGCCGCGTGAGTGATGAAGGTTTTCGGATCGTAAA<br>GCTCTGTTGTTAGGGAAGAACAAGTACCGTTCGAATAGGGCGGTACC<br>TTGACGGTACCTAACCAGAAAGCCACGGCTAACTACGTGCCAGCAGC<br>CGCGGTAATACGTAGGTGGCAAGCGTTGTCCGGAATTATTGGGCGTA<br>AAGGGCTCGCAGGCGGTTCCTTAAGTCTGATGTGAAAGCCCCCGGCT<br>CAACCGGGGAGGGTCATTGGAAACTGGGGAACTTGAGTGCAGAAGA<br>GGAGAGTGGAATTCCACGTGTAGCGGTGAAATGCGTAGAGATGTGGA<br>GGAACACCAGTGGCGAAGGCGACTCTCTGGTCTGTAACTGACGCTGA<br>GGAGCGAAAGCGTGGGGAGCGAACAGGATTAGATACCCTGGTAGTCC<br>ACGCCGTAAACGATGAGTGCTAAGTGTTAGGGGGTTTCCGCCCCTTAG<br>TGCTGCAGCTAACGCATTAAGCACTCCGCCTGGGGAGTACGGTCGCA<br>AGACTGAAACTCAAAGGAATTGACGGGGGCCCGCACAAGCGGTGGA<br>GCATGTGGTTTAATTCGAAGCAACGCGAAGAACCTTACCAGGTCTTG<br>ACATCCTCTGACAATCCTAGAGATAGGACGTCCCCTTCGGGGGCAGA<br>GTGACAGGTGGTGCATGGTTGTCGTCAGCTCGTGTCGTGAGATGTTGG<br>GTTAAGTCCCGCAACGAGCGCAACCCTTGATCTTAGTTGCCAGCATTC<br>AGTTGGGCACTCTAAGGTGACTGCCGGTGACAAACCGGAGGAAGGTG<br>GGGATGACGTCAAATCATCATGCCCCTTATGACCTGGGCTACACACGT<br>GCTACAATGGACAGAACAAAGGGCAGCGAAACCGCGAGGTTAAGCC<br>AATCCCACAAATCTGTTCTCAGTTCGGATCGCAGTCTGCAACTCGACT<br>GCGTGAAGCTGGAATCGCTAGTAATCGCGGATCAGCATGCCGC
F11	GATGGGAGCTTGCTCCCTGATGTTAGCGGCGGACGGGTGAGTAACAC<br>GTGGGTAACCTGCCTGTAAGACTGGGATAACTCCGGGAAACCGGGGC<br>TAATACCGGATGCTTGTTTGAACCGCATGGTTCAAACATAAAAGGTG<br>GCTTCGGCTACCACTTACAGATGGACCCGCGGCGCATTAGCTAGTTGG<br>TGAGGTAACGGCTCACCAAGGCAACGATGCGTAGCCGACCTGAGAGG<br>GTGATCGGCCACACTGGGACTGAGACACGGCCCAGACTCCTACGGGA<br>GGCAGCAGTAGGGAATTCTTCCGCAATGGACGAAAGTCTGACGGAGC<br>AACGCCGCGTGAGTGATGAAGGTTTTCGGATCGTAAAGCTCTGTTGTT

	AGGGAAGAACAAGTACCGTTCGAATAGGGCGGTACCTTGACGGTACC<br>TAACCAGAAAGCCACGGCTAACTACGTGCCAGCAGCCGCGGTAATAC<br>GTAGGTGGCAAGCGTTGTCCGGAATTATTGGGCGTAAAGGGCTCGCA<br>GGCGGTTCCTTAAGTCTGATGTGAAAGCCCCCGGCTCAACCGGGGAG<br>GGTCATTGGAAACTGGGGAACTTGAGTGCAGAAGAGGAGAGTGGAAT<br>TCCACGTGTAGCGGTGAAATGCGTAGAGATGTGGAGGAACACCAGTG<br>GCGAAGGCGACTCTCTGGTCTGTAACTGACGCTGAGGAGCGAAAGCG<br>TGGGGAGCGAACAGGATTAGATACCCTGGTAGTCCACGCCGTAAACG<br>ATGAGTGCTAAGTGTTAGGGGGTTTCCGCCCCTTAGTGCTGCAGCTAA<br>CGCATTAAGCACTCCGCCTGGGGAGTACGGTCGCAAGACTGAAACTC<br>AAAGGAATTGACGGGGGCCCGCACAAGCGGTGGAGCATGTGGTTTAA<br>TTCGAAGCAACGCGAAGAACCTTACCAGGTCTTGACATCCTCTGACA<br>ATCCTAGAGATAGGACGTCCCCTTCGGGGGCAGAGTGACAGGTGGTG<br>CATGGTTGTCGTCAGCTCGTGTCGTGAGATGTTGGGTTAAGTCCCGCA<br>ACGAGCGCAACCCTTGATCTTAGTTGCCAGCATTCAGTTGGGCACTCT<br>AAGGTGACTGCCGGTGACAAACCGGAGGAAGGTGGGGATGACGTCA<br>AATCATCATGCCCCTTATGACCTGGGCTACACACGTGCTACAATGGAC<br>AGAACAAAGGGCAGCGAAACCGCGAGGTTAAGCCAATCCCACAAAT<br>CTGTTCTCAGTTCGGATCGCAGTCTGCAACTCGACTGCGTGAAGCTGG<br>AATCGCTAGTAATCGCGGATCAGCATGCCGCGGTGAATACGTTCCCG<br>GGCCTTGTACACACCGCCCGTCACACCACGAGAGTTTGTAACACCCG<br>AAGTCGGTGAGGTAACCTTTATGGAGCC
S1	CGTGGGTAACCTGCCTGTAAGACTGGGATAACTCCGGGAAACCGGGG<br>CTAATACCGGATGGTTGTTTGAACCGCATGGTTCAAACATAAAAGGT<br>GGCTTCGGCTACCACTTACAGATGGACCCGCGGCGCATTAGCTAGTTG<br>GTGAGGTAACGGCTCACCAAGGCAACGATGCGTAGCCGACCTGAGAG<br>GGTGATCGGCCACACTGGGACTGAGACACGGCCCAGACTCCTACGGG<br>AGGCAGCAGTAGGGAATCTTCCGCAATGGACGAAAGTCTGACGGAGC<br>AACGCCGCGTGAGTGATGAAGGTTTTCGGATCGTAAAGCTCTGTTGTT<br>AGGGAAGAACAAGTACCGTTCGAATAGGGCGGTACCTTGACGGTACC<br>TAACCAGAAAGCCACGGCTAACTACGTGCCAGCAGCCGCGGTAATAC<br>GTAGGTGGCAAGCGTTGTCCGGAATTATTGGGCGTAAAGGGCTCGCA<br>GGCGGTTTCTTAAGTCTGATGTGAAAGCCCCCGGCTCAACCGGGGAG<br>GGTCATTGGAAACTGGGGAACTTGAGTGCAGAAGAGGAGAGTGGAAT<br>TCCACGTGTAGCGGTGAAATGCGTAGAGATGTGGAGGAACACCAGTG<br>GCGAAGGCGACTCTCTGGTCTGTAACTGACGCTGAGGAGCGAAAGCG<br>TGGGGAGCGAACAGGATTAGATACCCTGGTAGTCCACGCCGTAAACG<br>ATGAGTGCTAAGTGTTAGGGGGTTTCCGCCCCTTAGTGCTGCAGCTAA<br>CGCATTAAGCACTCCGCCTGGGGAGTACGGTCGCAAGACTGAAACTC<br>AAAGGAATTGACGGGGGCCCGCACAAGCGGTGGAGCATGTGGTTTAA<br>TTCGAAGCAACGCGAAGAACCTTACCAGGTCTTGACATCCTCTGACA<br>ATCCTAGAGATAGGACGTCCCCTTCGGGGGCAGAGTGACAGGTGGTG<br>CATGGTTGTCGTCAGCTCGTGTCGTGAGATGTTGGGTTAAGTCCCGCA<br>ACGAGCGCAACCCTTGATCTTAGTTGCCAGCATTCAGTTGGGCACTCT<br>AAGGTGACTGCCGGTGACAAACCGGAGGAAGGTGGGGATGACGTCA<br>AATCATCATGCCCCTTATGACCTGGGCTACACACGTGCTACAATGGAC<br>AGAACAAAGGGCAGCGAAACCGCGAGGTTAAGCCAATCCCACAAAT<br>CTGTTCTCAGTTCGGATCGCAGTCTGCAACTCGACTGCGTGAAGCTGG<br>AATCGCTAGTAATCGCGGATCAGCATGCCGCGGTGAATACGTTCCCG

	GGCCTTGTACACACCGCCCGTCACACCACGAGAGTTTGTAACACCCG<br>AAGTCGGT
S2	ACCCAGAATGTCCGGCGTGCCTAAATACATGCAAGTCGAGCGGACAG<br>ATGGGAGCTTGCTCCCTGATGTTAGCGGCGGACGGGTGAGTAACACG<br>TGGGTAACCTGCCTGTAAGACTGGGATAACTCCGGGAAACCGGGGCT<br>AATACCGGATGGTTGTTTGAACCGCATGGTTCAAACATAAAAGGTGG<br>CTTCGGCTACCACTTACAGATGGACCCGCGGCGCATTAGCTAGTTGGT<br>GAGGTAACGGCTCACCAAGGCAACGATGCGTAGCCGACCTGAGAGGG<br>TGATCGGCCACACTGGGACTGAGACACGGCCCAGACTCCTACGGGAG<br>GCAGCAGTAGGGAATCTTCCGCAATGGACGAAAGTCTGACGGAGCAA<br>CGCCGCGTGAGTGATGAAGGTTTTCGGATCGTAAAGCTCTGTTGTTAG<br>GGAAGAACAAGTACCGTTCGAATAGGGCGGTACCTTGACGGTACCTA<br>ACCAGAAAGCCACGGCTAACTACGTGCCAGCAGCCGCGGTAATACGT<br>AGGTGGCAAGCGTTGTCCGGAATTATTGGGCGTAAAGGGCTCGCAGG<br>CGGTTTCTTAAGTCTGATGTGAAAGCCCCCGGCTCAACCGGGGAGGG<br>TCATTGGAAACTGGGGAACTTGAGTGCAGAAGAGGAGAGTGGAATTC<br>CACGTGTAGCGGTGAAATGCGTAGAGATGTGGAGGAACACCAGTGGC<br>GAAGGCGACTCTCTGGTCTGTAACTGACGCTGAGGAGCGAAAGCGTG<br>GGGAGCGAACAGGATTAGATACCCTGGTAGTCCACGCCGTAAACGAT<br>GAGTGCTAAGTGTTAGGGGGTTTCCGCCCCTTAGTGCTGCAGCTAACG<br>CATTAAGCACTCCGCCTGGGGAGTACGGTCGCAAGACTGAAACTCAA<br>AGGAATTGACGGGGGCCCGCACAAGCGGTGGAGCATGTGGTTTAATT<br>CGAAGCAACGCGAAGAACCTTACCAGGTCTTGACATCCTCTGACAAT<br>CCTAGAGATAGGACGTCCCCTTCGGGGGCAGAGTGACAGGTGGTGCA<br>TGGTTGTCGTCAGCTCGTGTCGTGAGATGTTGGGTTAAGTCCCGCAAC<br>GAGCGCAACCCTTGATCTTAGTTGCCAGCATTCAGTTGGGCACTCTAA<br>GGTGACTGCCGGTGACAAACCGGAGGAAGGTGGGGATGACGTCAAAT<br>CATCATGCCCCTTATGACCTGGGCTACACACGTGCTACAATGGACAGA<br>ACAAAGGGCAGCGAAACCGCGAGGTTAAGCCAATCCCACAAATCTGT<br>TCTCAGTTCGGATCGCAGTCTGCAACTCGACTGCGTGAAGCTGGAATC<br>GCTAGTAATCGCGGATCAGCATGCCGCGGTGAATACGTTCCCGGGCC<br>TTGTACACACCGCCCGTCACACCACGAGAGTTTGTAACACCCGAAGTC<br>GGTGAGGTAACCTTTT
S3	AGTCGAGCGGACAGATGGGAGCTTGCTCCCTGATGTTAGCGGCGGAC<br>GGGTGAGTAACACGTGGGTAACCTGCCTGTAAGACTGGGATAACTCC<br>GGGAAACCGGGGCTAATACCGGATGGTTGTTTGAACCGCATGGTTCA<br>AACATAAAAGGTGGCTTCGGCTACCACTTACAGATGGACCCGCGGCG<br>CATTAGCTAGTTGGTGAGGTAATGGCTCACCAAGGCAACGATGCGTA<br>GCCGACCTGAGAGGGTGATCGGCCACACTGGGACTGAGACACGGCCC<br>AGACTCCTACGGGAGGCAGCAGTAGGGAATCTTCCGCAATGGACGAA<br>AGTCTGACGGAGCAACGCCGCGTGAGTGATGAAGGTTTTCGGATCGT<br>AAAGCTCTGTTGTTAGGGAAGAACAAGTACCGTTCGAATAGGGCGGT<br>ACCTTGACGGTACCTAACCAGAAAGCCACGGCTAACTACGTGCCAGC<br>AGCCGCGGTAATACGTAGGTGGCAAGCGTTGTCCGGAATTATTGGGC<br>GTAAAGGGCTCGCAGGCGGTTTCTTAAGTCTGATGTGAAAGCCCCCG<br>GCTCAACCGGGGAGGGTCATTGGAAACTGGGGAACTTGAGTGCAGAA<br>GAGGAGAGTGGAATTCCACGTGTAGCGGTGAAATGCGTAGAGATGTG<br>GAGGAACACCAGTGGCGAAGGCGACTCTCTGGTCTGTAACTGACGCT<br>GAGGAGCGAAAGCGTGGGGAGCGAACAGGATTAGATACCCTGGTAG<br>TCCACGCCGTAAACGATGAGTGCTAAGTGTTAGGGGGTTTCCGCCCCT

	TAGTGCTGCAGCTAACGCATTAAGCACTCCGCCTGGGGAGTACGGTC<br>GCAAGACTGAAACTCAAAGGAATTGACGGGGGCCCGCACAAGCGGT<br>GGAGCATGTGGTTTAATTCGAAGCAACGCGAAGAACCTTACCAGGTC<br>TTGACATCCTCTGACAATCCTAGAGATAGGACGTCCCCTTCGGGGGCA<br>GAGTGACAGGTGGTGCATGGTTGTCGTCAGCTCGTGTCGTGAGATGTT<br>GGGTTAAGTCCCGCAACGAGCGCAACCCTTGATCTTAGTTGCCAGCAT<br>TCAGTTGGGCACTCTAAGGTGACTGCCGGTGACAAACCGGAGGAAGG<br>TGGGGATGACGTCAAATCATCATGCCCCTTATGACCTGGGCTACACAC<br>GTGCTACAATGGACAGAACAAAGGGCAGCGAAACCGCGAGGTTAAG<br>CCAATCCCACAAATCTGTTCTCAGTTCGGATCGCAGTCTGCAACTCGA<br>CTGCGTGAAGCTGGAATCGCTAGTAATCGCGGATCAGCATGCCGCGG<br>TGAATACGTTCCCGGGCCTTGTACACACCGCCCGTCACACCACGAGA<br>GTTTGTAACACCCGAAGTCGGTGAGGTAA
S4	GAGCGGACAGATGGGAGCTTGCTCCCTGATGTTAGCGGCGGACGGGT<br>GAGTAACACGTGGGTAACCTGCCTGTAAGACTGGGATAACTCCGGGA<br>AACCGGGGCTAATACCGGATGCTTGTTTGAACCGCATGGTTCAAACAT<br>AAAAGGTGGCTTCGGCTACCACTTACAGATGGACCCGCGGCGCATTA<br>GCTAGTTGGTGAGGTAATGGCTCACCAAGGCAACGATGCGTAGCCGA<br>CCTGAGAGGGTGATCGGCCACACTGGGACTGAGACACGGCCCAGACT<br>CCTACGGGAGGCAGCAGTAGGGAATCTTCCGCAATGGACGAAAGTCT<br>GACGGAGCAACGCCGCGTGAGTGATGAAGGTTTTCGGATCGTAAAGC<br>TCTGTTGTTAGGGAAGAACAAGTACCGTTCGAATAGGGCGGTACCTT<br>GACGGTACCTAACCAGAAAGCCACGGCTAACTACGTGCCAGCAGCCG<br>CGGTAATACGTAGGTGGCAAGCGTTGTCCGGAATTATTGGGCGTAAA<br>GGGCTCGCAGGCGGTTTCTTAAGTCTGATGTGAAAGCCCCCGGCTCAA<br>CCGGGGAGGGTCATTGGAAACTGGGGAACTTGAGTGCAGAAGAGGA<br>GAGTGGAATTCCACGTGTAGCGGTGAAATGCGTAGAGATGTGGAGGA<br>ACACCAGTGGCGAAGGCGACTCTCTGGTCTGTAACTGACGCTGAGGA<br>GCGAAAGCGTGGGGAGCGAACAGGATTAGATACCCTGGTAGTCCACG<br>CCGTAAACGATGAGTGCTAAGTGTTAGGGGGTTTCCGCCCCTTAGTGC<br>TGCAGCTAACGCATTAAGCACTCCGCCTGGGGAGTACGGTCGCAAGA<br>CTGAAACTCAAAGGAATTGACGGGGGCCCGCACAAGCGGTGGAGCAT<br>GTGGTTTAATTCGAAGCAACGCGAAGAACCTTACCAGGTCTTGACATC<br>CTCTGACAATCCTAGAGATAGGACGTCCCCTTCGGGGGCAGAGTGAC<br>AGGTGGTGCATGGTTGTCGTCAGCTCGTGTCGTGAGATGTTGGGTTAA<br>GTCCCGCAACGAGCGCAACCCTTGATCTTAGTTGCCAGCATTCAGTTG<br>GGCACTCTAAGGTGACTGCCGGTGACAAACCGGAGGAAGGTGGGGAT<br>GACGTCAAATCATCATGCCCCTTATGACCTGGGCTACACACGTGCTAC<br>AATGGACAGAACAAAGGGCAGCGAAACCGCGAGGTTAAGCCAATCC<br>CACAAATCTGTTCTCAGTTCGGATCGCAGTCTGCAACTCGACTGCGTG<br>AAGCTGGAATCGCTAGTAATCGCGGATCAGCATGCCGCGGTGAATAC<br>GTTCCCGGGCCTTGTACACACCGCCCGTCACACCACGAGAGTTTGTAA<br>CACCCGAAGTCGGTGAGGTAACCTTTTAGG
S5	GTCGAGCGAACTGATTAGAAGCTTGCTTCTATGACGTTAGCGGCGGA<br>CGGGTGAGTAACACGTGGGCAACCTGCCTGTAAGACTGGGATAACTT<br>CGGGAAACCGAAGCTAATACCGGATAGGATCTTCTCCTTCATGGGAG<br>ATGATTGAAAGATGGTTTCGGCTATCACTTACAGATGGGCCCGCGGTG<br>CATTAGCTAGTTGGTGAGGTAACGGCTCACCAAGGCAACGATGCATA<br>GCCGACCTGAGAGGGTGATCGGCCACACTGGGACTGAGACACGGCCC<br>AGACTCCTACGGGAGGCAGCAGTAGGGAATCTTCCGCAATGGACGAA

	AGTCTGACGGAGCAACGCCGCGTGAGTGATGAAGGCTTTCGGGTCGT<br>AAAACTCTGTTGTTAGGGAAGAACAAGTACAAGAGTAACTGCTTGTA<br>CCTTGACGGTACCTAACCAGAAAGCCACGGCTAACTACGTGCCAGCA<br>GCCGCGGTAATACGTAGGTGGCAAGCGTTATCCGGAATTATTGGGCG<br>TAAAGCGCGCGCAGGCGGTTTCTTAAGTCTGATGTGAAAGCCCACGG<br>CTCAACCGTGGAGGGTCATTGGAAACTGGGGAACTTGAGTGCAGAAG<br>AGAAAAGCGGAATTCCACGTGTAGCGGTGAAATGCGTAGAGATGTGG<br>AGGAACACCAGTGGCGAAGGCGGCTTTTTGGTCTGTAACTGACGCTG<br>AGGCGCGAAAGCGTGGGGAGCAAACAGGATTAGATACCCTGGTAGTC<br>CACGCCGTAAACGATGAGTGCTAAGTGTTAGAGGGTTTCCGCCCTTTA<br>GTGCTGCAGCTAACGCATTAAGCACTCCGCCTGGGGAGTACGGTCGC<br>AAGACTGAAACTCAAAGGAATTGACGGGGGCCCGCACAAGCGGTGG<br>AGCATGTGGTTTAATTCGAAGCAACGCGAAGAACCTTACCAGGTCTT<br>GACATCCTCTGACAACTCTAGAGATAGAGCGTTCCCCTTCGGGGGAC<br>AGAGTGACAGGTGGTGCATGGTTGTCGTCAGCTCGTGTCGTGAGATGT<br>TGGGTTAAGTCCCGCAACGAGCGCAACCCTTGATCTTAGTTGCCAGCA<br>TTTAGTTGGGCACTCTAAGGTGACTGCCGGTGACAAACCGGAGGAAG<br>GTGGGGATGACGTCAAATCATCATGCCCCTTATGACCTGGGCTACACA<br>CGTGCTACAATGGATGGTACAAAGGGCTGCAAGACCGCGAGGTCAAG<br>CCAATCCCATAAAACCATTCTCAGTTCGGATTGTAGGCTGCAACTCGC<br>CTACATGAAGCTGGAATCGCTAGTAATCGCGGATCAGCATGCCGCGG<br>TGAATACGTTCCCGGGCCTTGTACACACCGCCCGTCACACCACGAGA<br>GTTTGTAACACCCGAAGTCGGTGGAGTAAACCGTAAAGGAGCT
S6	CGAAGCGGACAGATGGGAGCTTGCTCCCTGATGTTAGCGGCGGACGG<br>GTGAGTAACACGTGGGTAACCTGCCTGTAAGACTGGGATAACTCCGG<br>GAAACCGGGGCTAATACCGGATGGTTGTTTGAACCGCATGGTTCAAA<br>CATAAAAGGTGGCTTCGGCTACCACTTACAGATGGACCCGCGGCGCA<br>TTAGCTAGTTGGTGAGGTAACGGCTCACCAAGGCAACGATGCGTAGC<br>CGACCTGAGAGGGTGATCGGCCACACTGGGACTGAGACACGGCCCAG<br>ACTCCTACGGGAGGCAGCAGTAGGGAATCTTCCGCAATGGACGAAAG<br>TCTGACGGAGCAACGCCGCGTGAGTGATGAAGGTTTTCGGATCGTAA<br>AGCTCTGTTGTTAGGGAAGAACAAGTACCGTTCGAATAGGGCGGTAC<br>CTTGACGGTACCTAACCAGAAAGCCACGGCTAACTACGTGCCAGCAG<br>CCGCGGTAATACGTAGGTGGCAAGCGTTGTCCGGAATTATTGGGCGT<br>AAAGGGCTCGCAGGCGGTTTCTTAAGTCTGATGTGAAAGCCCCCGGC<br>TCAACCGGGGAGGGTCATTGGAAACTGGGGAACTTGAGTGCAGAAGA<br>GGAGAGTGGAATTCCACGTGTAGCGGTGAAATGCGTAGAGATGTGGA<br>GGAACACCAGTGGCGAAGGCGACTCTCTGGTCTGTAACTGACGCTGA<br>GGAGCGAAAGCGTGGGGAGCGAACAGGATTAGATACCCTGGTAGTCC<br>ACGCCGTAAACGATGAGTGCTAAGTGTTAGGGGGTTTCCGCCCCTTAG<br>TGCTGCAGCTAACGCATTAAGCACTCCGCCTGGGGAGTACGGTCGCA<br>AGACTGAAACTCAAAGGAATTGACGGGGGCCCGCACAAGCGGTGGA<br>GCATGTGGTTTAATTCGAAGCAACGCGAAGAACCTTACCAGGTCTTG<br>ACATCCTCTGACAATCCTAGAGATAGGACGTCCCCTTCGGGGGCAGA<br>GTGACAGGTGGTGCATGGTTGTCGTCAGCTCGTGTCGTGAGATGTTGG<br>GTTAAGTCCCGCAACGAGCGCAACCCTTGATCTTAGTTGCCAGCATTC<br>AGTTGGGCACTCTAAGGTGACTGCCGGTGACAAACCGGAGGAAGGTG<br>GGGATGACGTCAAATCATCATGCCCCTTATGACCTGGGCTACACACGT<br>GCTACAATGGACAGAACAAAGGGCAGCGAAACCGCGAGGTTAAGCC<br>AATCCCACAAATCTGTTCTCAGTTCGGATCGCAGTCTGCAACTCGACT

	GCGTGAAGCTGGAATCGCTAGTAATCGCGGATCAGCATGCCGCGGTG<br>AATACGTTCCCGGGCCTTGTACACACCGCCCGTCACACCACGAGAGT
S7	AATACATGCAGTCGAGCGGACAGATGGGAGCTTGCTCCCTGATGTTA<br>GCGGCGGACGGGTGAGTAACACGTGGGTAACCTGCCTGTAAGACTGG<br>GATAACTCCGGGAAACCGGGGCTAATACCGGATGGTTGTTTGAACCG<br>CATGGTTCAAACATAAAAGGTGGCTTCGGCTACCACTTACAGATGGA<br>CCCGCGGCGCATTAGCTAGTTGGTGAGGTAACGGCTCACCAAGGCAA<br>CGATGCGTAGCCGACCTGAGAGGGTGATCGGCCACACTGGGACTGAG<br>ACACGGCCCAGACTCCTACGGGAGGCAGCAGTAGGGAATCTTCCGCA<br>ATGGACGAAAGTCTGACGGAGCAACGCCGCGTGAGTGATGAAGGTTT<br>TCGGATCGTAAAGCTCTGTTGTTAGGGAAGAACAAGTACCGTTCGAA<br>TAGGGCGGTACCTTGACGGTACCTAACCAGAAAGCCACGGCTAACTA<br>CGTGCCAGCAGCCGCGGTAATACGTAGGTGGCAAGCGTTGTCCGGAA<br>TTATTGGGCGTAAAGGGCTCGCAGGCGGTTTCTTAAGTCTGATGTGAA<br>AGCCCCCGGCTCAACCGGGGAGGGTCATTGGAAACTGGGGAACTTGA<br>GTGCAGAAGAGGAGAGTGGAATTCCACGTGTAGCGGTGAAATGCGTA<br>GAGATGTGGAGGAACACCAGTGGCGAAGGCGACTCTCTGGTCTGTAA<br>CTGACGCTGAGGAGCGAAAGCGTGGGGAGCGAACAGGATTAGATAC<br>CCTGGTAGTCCACGCCGTAAACGATGAGTGCTAAGTGTTAGGGGGTTT<br>CCGCCCCTTAGTGCTGCAGCTAACGCATTAAGCACTCCGCCTGGGGAG<br>TACGGTCGCAAGACTGAAACTCAAAGGAATTGACGGGGGCCCGCACA<br>AGCGGTGGAGCATGTGGTTTAATTCGAAGCAACGCGAAGAACCTTAC<br>CAGGTCTTGACATCCTCTGACAATCCTAGAGATAGGACGTCCCCTTCG<br>GGGGCAGAGTGACAGGTGGTGCATGGTTGTCGTCAGCTCGTGTCGTG<br>AGATGTTGGGTTAAGTCCCGCAACGAGCGCAACCCTTGATCTTAGTTG<br>CCAGCATTCAGTTGGGCACTCTAAGGTGACTGCCGGTGACAAACCGG<br>AGGAAGGTGGGGATGACGTCAAATCATCATGCCCCTTATGACCTGGG<br>CTACACACGTGCTACAATGGACAGAACAAAGGGCAGCGAAACCGCG<br>AGGTTAAGCCAATCCCACAAATCTGTTCTCAGTTCGGATCGCAGTCTG<br>CAACTCGACTGCGTGAAGCTGGAATCGCTAGTAATCGCGGATCAGCA<br>TGCCGCGGTGAATACGTTCCCGGGCCTTGTACACACCGCCCGTCACAC<br>CACGAGAGTTTGTAACACCCGAAGTCGGTGAGGTAACCTTTTAGGA
S8	TCGAGCGAACTGATTAGAAGCTTGCTTCTATGACGTTAGCGGCGGAC<br>GGGTGAGTAACACGTGGGCAACCTGCCTGTAAGACTGGGATAACTTC<br>GGGAAACCGAAGCTAATACCGGATAGGATCTTCTCCTTCATGGGAGA<br>TGATTGAAAGATGGTTTCGGCTATCACTTACAGATGGGCCCGCGGTGC<br>ATTAGCTAGTTGGTGAGGTAACGGCTCACCAAGGCAACGATGCATAG<br>CCGACCTGAGAGGGTGATCGGCCACACTGGGACTGAGACACGGCCCA<br>GACTCCTACGGGAGGCAGCAGTAGGGAATCTTCCGCAATGGACGAAA<br>GTCTGACGGAGCAACGCCGCGTGAGTGATGAAGGCTTTCGGGTCGTA<br>AAACTCTGTTGTTAGGGAAGAACAAGTACAAGAGTAACTGCTTGTAC<br>CTTGACGGTACCTAACCAGAAAGCCACGGCTAACTACGTGCCAGCAG<br>CCGCGGTAATACGTAGGTGGCAAGCGTTATCCGGAATTATTGGGCGT<br>AAAGCGCGCGCAGGCGGTTTCTTAAGTCTGATGTGAAAGCCCACGGC<br>TCAACCGTGGAGGGTCATTGGAAACTGGGGAACTTGAGTGCAGAAGA<br>GAAAAGCGGAATTCCACGTGTAGCGGTGAAATGCGTAGAGATGTGGA<br>GGAACACCAGTGGCGAAGGCGGCTTTTTGGTCTGTAACTGACGCTGA<br>GGCGCGAAAGCGTGGGGAGCAAACAGGATTAGATACCCTGGTAGTCC<br>ACGCCGTAAACGATGAGTGCTAAGTGTTAGAGGGTTTCCGCCCTTTAG<br>TGCTGCAGCTAACGCATTAAGCACTCCGCCTGGGGAGTACGGTCGCA

	AGACTGAAACTCAAAGGAATTGACGGGGGCCCGCACAAGCGGTGGA<br>GCATGTGGTTTAATTCGAAGCAACGCGAAGAACCTTACCAGGTCTTG<br>ACATCCTCTGACAACTCTAGAGATAGAGCGTTCCCCTTCGGGGGACA<br>GAGTGACAGGTGGTGCATGGTTGTCGTCAGCTCGTGTCGTGAGATGTT<br>GGGTTAAGTCCCGCAACGAGCGCAACCCTTGATCTTAGTTGCCAGCAT<br>TTAGTTGGGCACTCTAAGGTGACTGCCGGTGACAAACCGGAGGAAGG<br>TGGGGATGACGTCAAATCATCATGCCCCTTATGACCTGGGCTACACAC<br>GTGCTACAATGGATGGTACAAAGGGCTGCAAGACCGCGAGGTCAAGC<br>CAATCCCATAAAACCATTCTCAGTTCGGATTGTAGGCTGCAACTCGCC<br>TACATGAAGCTGGAATCGCTAGTAATCGCGGATCAGCATGCCGCGGT<br>GAATACGTTCCCGGGCCTTGTACACACCGCCCGTCACACCACGAGAG<br>TTTGTAACACCCGAAGTCGGTGGAGTAACCGTAAGGAG
A1	GAGCTTGCTCCCGGATGTTAGCGGCGGACGGGTGAGTAACACGTGGG<br>TAACCTGCCTGTAAGACTGGGATAACTCCGGGAAACCGGAGCTAATA<br>CCGGATAGTTCCTTGAACCGCATGGTTCAAGGATGAAAGACGGTTTC<br>GGCTGTCACTTACAGATGGACCCGCGGCGCATTAGCTAGTTGGTGAG<br>GTAACGGCTCACCAAGGCGACGATGCGTAGCCGACCTGAGAGGGTGA<br>TCGGCCACACTGGGACTGAGACACGGCCCAGACTCCTACGGGAGGCA<br>GCAGTAGGGAATCTTCCGCAATGGACGAAAGTCTGACGGAGCAACGC<br>CGCGTGAGTGATGAAGGTTTTCGGATCGTAAAGCTCTGTTGTTAGGGA<br>AGAACAAGTGCAAGAGTAACTGCTTGCACCTTGACGGTACCTAACCA<br>GAAAGCCACGGCTAACTACGTGCCAGCAGCCGCGGTAATACGTAGGT<br>GGCAAGCGTTGTCCGGAATTATTGGGCGTAAAGGGCTCGCAGGCGGT<br>TTCTTAAGTCTGATGTGAAAGCCCCCGGCTCAACCGGGGAGGGTCATT<br>GGAAACTGGGAAACTTGAGTGCAGAAGAGGAGAGTGGAATTCCACGT<br>GTAGCGGTGAAATGCGTAGAGATGTGGAGGAACACCAGTGGCGAAG<br>GCGACTCTCTGGTCTGTAACTGACGCTGAGGAGCGAAAGCGTGGGGA<br>GCGAACAGGATTAGATACCCTGGTAGTCCACGCCGTAAACGATGAGT<br>GCTAAGTGTTAGGGGGTTTCCGCCCCTTAGTGCTGCAGCTAACGCATT<br>AAGCACTCCGCCTGGGGAGTACGGTCGCAAGACTGAAACTCAAAGGA<br>ATTGACGGGGGCCCGCACAAGCGGTGGAGCATGTGGTTTAATTCGAA<br>GCAACGCGAAGAACCTTACCAGGTCTTGACATCCTCTGACAACCCTA<br>GAGATAGGGCTTTCCCTTCGGGGACAGAGTGACAGGTGGTGCATGGT<br>TGTCGTCAGCTCGTGTCGTGAGATGTTGGGTTAAGTCCCGCAACGAGC<br>GCAACCCTTGATCTTAGTTGCCAGCATTCAGTTGGGCACTCTAAGGTG<br>ACTGCCGGTGACAAACCGGAGGAAGGTGGGGATGACGTCAAATCATC<br>ATGCCCCTTATGACCTGGGCTACACACGTGCTACAATGGACAGAACA<br>AAGGGCTGCGAGACCGCAAGGTTTAGCCAATCCCACAAATCTGTTCT<br>CAGTTCGGATCGCAGTCTGCAACTCGACTGCGTGAAGCTGGAATCGCT<br>AGTAATCGCGGATCAGCATGCCGCGGTGAATACGTTCCCGGGCCTTGT<br>ACACACCGCCCGTCACACCACGAGAGTTTGCAACACCCGAAGTCGGT<br>GAGG
A2	CCTGAGAGGGTGGATCGGCCACACTGGGACTGAGACACGGCCCAGAC<br>TCCTACGGGAGGCAGCAGTAGGGAATCTTCCGCAATGGACGAAAGTC<br>TGACGGAGCAACGCCGCGTGAGTGATGAAGGTTTTCGGATCGTAAAG<br>CTCTGTTGTTAGGGAAGAACAAGTGCGAGAGTAACTGCTCGCACCTT<br>GACGGTACCTAACCAGAAAGCCACGGCTAACTACGTGCCAGCAGCCG<br>CGGTAATACGTAGGTGGCAAGCGTTGTCCGGAATTATTGGGCGTAAA<br>GGGCTCGCAGGCGGTTTCTTAAGTCTGATGTGAAAGCCCCCGGCTCAA<br>CCGGGGAGGGTCATTGGAAACTGGGAAACTTGAGTGCAGAAGAGGA

	GAGTGGAATTCCACGTGTAAGCGGTGAAAATGCGTAGAGATGTGGAG<br>GAAACACCAGTGGCGAAGGCGACTCTCTGGTCTGTAACTGACGCTGA<br>GGAGCGAAAGCGTGGGGAGCGAAACAGGATTAGATACCCTGGTAG<br>TCCACGCCGTAAACGATGAGTGCTAAGTGTTAGGGGGTTTCCGCCCCT<br>TAGTGCTGCAGCTAACGCATTAAGCACTCCGCCTGGGGAGTACGGTC<br>GCAAGACTGAAACTCAAAGGAATTGACGGGGGCCCGCACAAGCGGT<br>GGAGCATGTGGTTTAATTCGAAGCAACGCGAAGAACCTTACCAGGTC<br>TTGACATCCTCTGACAACCCTAGAGATAGGGCTTTCCCTTCGGGGACA<br>GAGTGACAGGTGGTGCATGGTTGTCGTCAGCTCGTGTCGTGAGATGTT<br>GGGTTAAGTCCCGCAACGAGCGCAACCCTTGATCTTAGTTGCCAGCAT<br>TCAGTTGGGCACTCTAAGGTGACTGCCGGTGACAAACCGGAGGAAGG<br>TGGGGATGACGTCAAATCATCATGCCCCTTATGACCTGGGCTACACAC<br>GTGCTACAATGGACAGAACAAAGGGCTGCAAGACCGCAAGGTTTAGC<br>CAATCCCATAAATCTGTTCTCAGTTCGGATCGCAGTCTGCAACTCGAC<br>TGCGTGAAGCTGGAATCGCTAGTAATCGCGGATCAGCATGCCGCGGT<br>GAATACGTTCCCGGGCCTTGTACACACCGCCCGTCACACCACGAGAG<br>TTTGCAACACCCGAAGTCGGTGAGGTAAC
A3	AAGTCGAGCGGACAGAAGGGAGCTTGCTCCCGGATGTTAGCGGCGGA<br>CGGGTGAGTAACACGTGGGTAACCTGCCTGTAAGACTGGGATAACTC<br>CGGGAAACCGGAGCTAATACCGGATAGTTCCTTGAACCGCATGGTTC<br>AAGGATGAAAGACGGTTTCGGCTGTCACTTACAGATGGACCCGCGGC<br>GCATTAGCTAGTTGGTGGGGTAATGGCTCACCAAGGCGACGATGCGT<br>AGCCGACCTGAGAGGGTGATCGGCCACACTGGGACTGAGACACGGCC<br>CAGACTCCTACGGGAGGCAGCAGTAGGGAATCTTCCGCAATGGACGA<br>AAGTCTGACGGAGCAACGCCGCGTGAGTGATGAAGGTTTTCGGATCG<br>TAAAGCTCTGTTGTTAGGGAAGAACAAGTGCGAGAGTAACTGCTCGC<br>ACCTTGACGGTACCTAACCAGAAAGCCACGGCTAACTACGTGCCAGC<br>AGCCGCGGTAATACGTAGGTGGCAAGCGTTGTCCGGAATTATTGGGC<br>GTAAAGGGCTCGCAGGCGGTTTCTTAAGTCTGATGTGAAAGCCCCCG<br>GCTCAACCGGGGAGGGTCATTGGAAACTGGGAAACTTGAGTGCAGAA<br>GAGGAGAGTGGAATTCCACGTGTAGCGGTGAAATGCGTAGAGATGTG<br>GAGGAACACCAGTGGCGAAGGCGACTCTCTGGTCTGTAACTGACGCT<br>GAGGAGCGAAAGCGTGGGGAGCGAACAGGATTAGATACCCTGGTAG<br>TCCACGCCGTAAACGATGAGTGCTAAGTGTTAGGGGGTTTCCGCCCCT<br>TAGTGCTGCAGCTAACGCATTAAGCACTCCGCCTGGGGAGTACGGTC<br>GCAAGACTGAAACTCAAAGGAATTGACGGGGGCCCGCACAAGCGGT<br>GGAGCATGTGGTTTAATTCGAAGCAACGCGAAGAACCTTACCAGGTC<br>TTGACATCCTCTGACAACCCTAGAGATAGGGCTTTCCCTTCGGGGACA<br>GAGTGACAGGTGGTGCATGGTTGTCGTCAGCTCGTGTCGTGAGATGTT<br>GGGTTAAGTCCCGCAACGAGCGCAACCCTTGATCTTAGTTGCCAGCAT<br>TCAGTTGGGCACTCTAAGGTGACTGCCGGTGACAAACCGGAGGAAGG<br>TGGGGATGACGTCAAATCATCATGCCCCTTATGACCTGGGCTACACAC<br>GTGCTACAATGGACAGAACAAAGGGCTGCAAGACCGCAAGGTTTAGC<br>CAATCCCATAAATCTGTTCTCAGTTCGGATCGCAGTCTGCAACTCGAC<br>TGCGTGAAGCTGGAATCGCTAGTAATCGCGGATCAGCATGCCGCGGT<br>GAATACGTTCCCGGGCCTTGTACACACCGCCCGTCACACCACGAGAG<br>TTTGCAACACCCGAAGTCGGTGAGGTAA

Supplementary Fig. S1. The representative images of agar plate assays. (A) result of enzymatic activity, **(B)** result of antibacterial activity. Enzymatic and antibacterial activities were calculated as the clearance zone size (mm) divided by the colony size (mm). Redline, clearance zone size (mm). Blueline, colony size (mm).

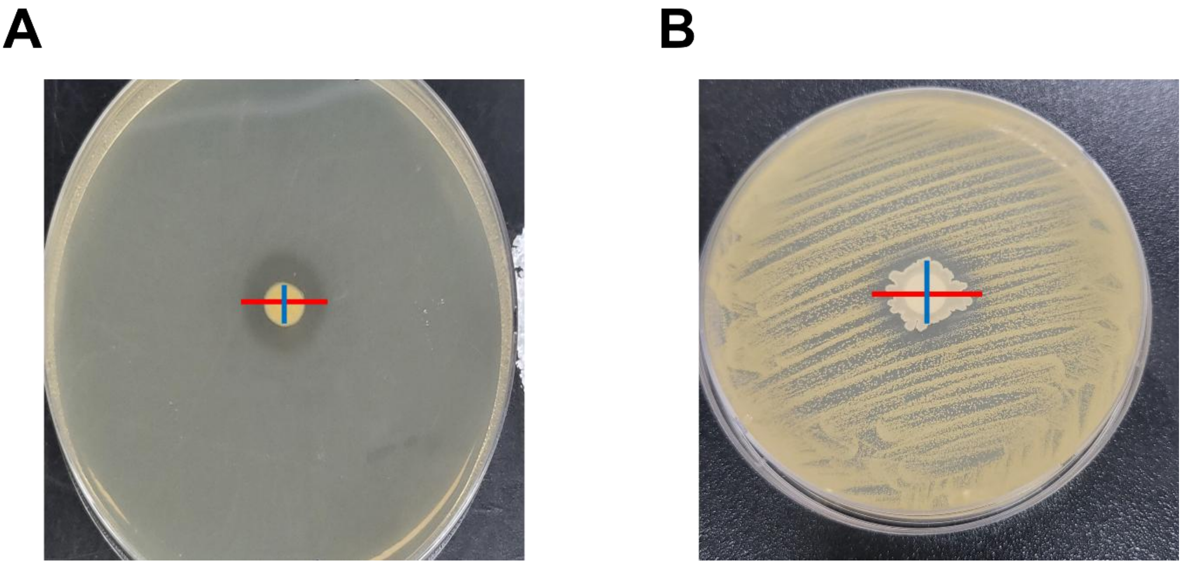

Supplement: Supplementary file 1 [file jmb-35-e2411070-supple.pdf]
